# Exercise-induced systemic and tumor-microenvironment adaptations regulating chemokine-guided immune-cell trafficking

**DOI:** 10.1007/s10238-026-02315-z

**Published:** 2026-09-01

**Authors:** Amir Hossein Ahmadi Hekmatikar, Aliakbar Yousefi-Ahmadipour, Diego Fernández-Lázaro

**Affiliations:** 1 Department of Sport Science, Faculty of Humanities, Tarbiat Modares University, Tehran, Iran; 2 Immunology of Infectious Diseases Research Center, Research Institute of Basic Medical Sciences, Rafsanjan University of Medical Sciences, Rafsanjan, Iran; 3 Molecular Medicine Research Center, Research Institute of Basic Medical Sciences, Rafsanjan University of Medical Sciences, Rafsanjan, Iran; 4 Department of Laboratory Sciences, Faculty of Paramedicine, Rafsanjan University of Medical Sciences, Rafsanjan, Iran; 5 Cancer and Stem Cell Research Laboratory, Faculty of Paramedicine, Rafsanjan University of Medical Sciences, Rafsanjan, Iran; 6 Neurobiology Research Group, Faculty of Medicine, University of Valladolid, Valladolid, 47005 Spain; 7 Area of Histology, Faculty of Health Sciences, University of Valladolid, Campus at Soria, Soria, 42004 Spain; 8 Consolidated Research Group ENSADE (Envejecimiento, Neurociencia, Salud y Desarrollo), León Biosanitary Research Institute (IBIOLEÓN), León, 24071 Spain

**Keywords:** Exercise Oncology, Tumor Microenvironment, Chemokine Gradients, Immune Cell Homing, T-cell Trafficking, Vascular Normalization, Immunotherapy

## Abstract

Exercise is increasingly recognized as a biologically active intervention in cancer, yet its role in shaping the chemokine landscape of the tumor microenvironment remains incompletely defined. Importantly, immune-cell mobilization in the circulation does not necessarily translate into productive tumor infiltration, because effective homing depends on chemokine availability, endothelial permissiveness, stromal architecture, metabolic state, and spatial organization within the tumor microenvironment. In this review, we propose that exercise functions as a multiscale physiological regulator that may influence chemokine production, presentation, accessibility, and interpretation across transcriptional, vascular, stromal, and metabolic levels. We focus on three major axes relevant to immune-cell trafficking: CXCL9/10/11–CXCR3, CCL5–CCR5, and CXCL12–CXCR4. The CXCL9/10/11–CXCR3 pathway appears to provide the strongest mechanistic link between exercise-conditioned inflammatory signaling and cytotoxic T-cell recruitment. By contrast, CCL5–CCR5 is highly context dependent and may support either effector-cell trafficking or suppressive myeloid recruitment. CXCL12–CXCR4 likely represents a dominant retention and exclusion pathway associated with stromal sequestration, hypoxia, and vascular dysfunction. Although available evidence supports exercise-induced changes in systemic inflammatory mediators and selected circulating chemokines, direct evidence for reproducible exercise-driven remodeling of intratumoral chemokine gradients remains limited. Accordingly, exercise should not be viewed as a universal chemokine-normalizing intervention, but rather as a context-dependent modulator of immune accessibility that may be most effective when combined with immunotherapy, vascular normalization, or stromal-targeting strategies. Future work will require spatially resolved tumor analyses, rigorous exercise-dose reporting, serial sampling, and causal testing of exerkine–chemokine interactions.

**Supplementary Information:**

The online version contains supplementary material available at https://doi.org/10.1007/s10238-026-02315-z.

## Highlights

Exercise mobilizes circulating immune cells, but mobilization alone does not guarantee tumor infiltration.

Effective immune homing depends on chemokine gradients, endothelial access, stromal architecture, and metabolic conditions.

The CXCL9/10/11–CXCR3 axis is the strongest candidate pathway linking exercise-conditioned inflammation to effector T-cell recruitment.

CCL5–CCR5 is context dependent and may support either anti-tumor trafficking or suppressive myeloid recruitment.

CXCL12–CXCR4 likely functions as a retention and exclusion pathway within stromal niches.

Exercise effects are likely dose-dependent, time-dependent, and tumor-context dependent rather than universally beneficial.

Future studies must prioritize spatial intratumoral measurements, not just serum biomarkers.

**Supplementary Information:**

The online version contains supplementary material available at https://doi.org/10.1007/s10238-026-02315-z.

## Introduction

 Exercise is increasingly recognized as a systemic modulator of host immunity and a potential non-pharmacological adjuvant in oncology [1–7]. Accumulating evidence indicates that physical activity influences antitumor immunosurveillance by altering circulating leukocyte dynamics, cytokine profiles, metabolic signaling, vascular function, and stromal biology [1–7]. However, despite these pleiotropic systemic adaptations, translating exercise-induced immune activation into effective, localized antitumor immunity remains an unresolved challenge [1–7]. The critical bottleneck in exercise oncology is not merely whether physical exertion mobilizes immune effectors into the peripheral circulation [1–7], but whether it successfully orchestrates the recruitment, homing, and functional positioning of these effectors within the immunosuppressive tumor microenvironment (TME) [1–3, 5, 6]. Among the cytotoxic populations essential for tumor clearance are CD8^+^cytotoxic T lymphocytes (CTLs), CD4^+^, CD8^+^cell subsets, and natural killer (NK) cells [5, 6]. While these populations represent the primary effector arms of adaptive and innate antitumor immunity, their therapeutic efficacy is strictly contingent upon successful trafficking into the tumor parenchyma and sustained engagement with malignant cells [5, 6]. In many solid tumors—particularly those exhibiting an “immune-excluded” or “immune-desert” phenotype—intratumoral infiltration is severely restricted despite robust systemic mobilization or marginal accumulation at the invasive border. This clinical disconnect demonstrates that the primary barrier to immune-mediated tumor control is often not systemic immune deficiency, but rather a localized trafficking defect characterized by impaired homing and physical exclusion [5, 6].

The failure of CD4^+^, CD8^+^, and NK cells to infiltrate the tumor core is driven by a multi-layered trafficking barrier engineered by the TME [8, 9]. Endothelial cell activation, chemokine gradient disorganization, extracellular matrix (ECM) remodeling, immunosuppressive stromal cells, and elevated interstitial fluid pressure (IFP) cooperatively dictate leukocyte extravasation and migration [8, 9]. In immune-excluded tumors, chemokine expression is often spatially distorted or silenced, endothelial cell-adhesion molecules are downregulated, and the stroma presents a dense mechanical barrier [10]. These alterations collectively prevent effector cells from accessing tumor nests, often redirecting them to the stroma where they are rendered dysfunctional [10]. Chemokine pathways, specifically the CXCL9/10/11–CXCR3, CCL5–CCR5, and CXCL12–CXCR4 axes, serve as the primary molecular cues directing the homing of T and NK cells [11]. Epigenetic silencing or proteolytic degradation of attractant chemokines, combined with the overexpression of decoy receptors or chemokine-sequestering stromal factors, disrupts these essential gradients [11] Concurrently, dysfunctional tumor angiogenesis—characterized by structural vessel instability and altered shear stress—limits leukocyte adhesion, while dense collagen deposition by cancer-associated fibroblasts (CAFs) physically impedes direct migration, establishing a dual biochemical and mechanical blockade to immune infiltration [11]. Lymphatic vessels should also be considered as integral components of this barrier and transport network [12, 13]. In tumors, lymphatic remodeling and lymphangiogenesis regulate interstitial fluid drainage, local pressure gradients, antigen delivery, dendritic-cell trafficking, lymphocyte recirculation, and communication with tumor-draining lymph nodes [14]. This compartment is therefore not merely passive conduct but an active regulator of inflammatory tone and antitumor immunity [15]. This is particularly relevant in the context of the CCL19/CCL21–CCR7 axis, which coordinates dendritic-cell and T-cell migration across lymphatic and nodal niches.

These observations raise a pivotal mechanistic question: can exercise training reprogram the TME to restore functional immune-cell homing? Physical exercise exerts direct mechanical and metabolic forces on both healthy and malignant tissues. Shearing forces from increased blood flow, transient hypoxia, and systemic muscle-tumor cross-talk mediated by myokines (e.g., IL-15) and exerkines have been shown to modulate vascular remodeling, ECM architecture, and local cytokine production [1–7]. These exercise-induced alterations possess the potential to reset the chemokine signaling balance, upregulate adhesion molecules on the tumor endothelium, and normalize tumor vasculature, thereby facilitating the transition of immune cells from circulation to the tumor core [1–7]. Despite these insights, the molecular mechanisms linking physical exercise to immune trafficking remain fragmented. While previous literature has extensively documented systemic immune cell mobilization and descriptive changes in tumor regression, how exercise specifically modulates the molecular and physical dynamics of intratumoral homing remains a critical knowledge gap. A comprehensive, mechanically focused framework that links exercise-induced systemic signals to chemokine gradient restoration, vascular normalization, and the migration of CD4^+^, CD8^+^, and NK cells is required to advance the field. This review synthesizes current evidence at the intersection of exercise physiology and tumor immunology, focusing on how exercise-induced biochemical and biophysical adaptations can be leveraged to dismantle the barriers of the TME and re-establish immune cell homing. The evidence base for this narrative review was assembled through a structured search of PubMed/MEDLINE, Scopus, and Web of Science, followed by manual screening of reference lists and semantically related review articles. Search domains, inclusion criteria, and methodological details are reported in Appendix (Sect. 1, Table S1 and Table S2). The conceptual framework underlying the review is summarized in Figure S1.

## The molecular syntax of chemokine gradients in the TME

The spatiotemporal organization of the tumor microenvironment (TME) is shaped in large part by chemokine-directed communication networks that regulate the recruitment, positioning, retention, and recirculation of immune and stromal cell populations [16, 17]. The principles governing chemokine structure, receptor engagement, matrix association, and immune-cell positioning are well established across inflammatory and tumor settings. However, the extent to which exercise remodels these chemokine systems directly within human tumors remains incompletely defined. Therefore, the established biology of chemokine signaling should be distinguished from the exercise-related mechanisms discussed below, many of which represent biologically plausible but experimentally incompletely validated hypotheses [16, 17]. Chemokines are small secreted proteins, typically 8–15 kDa in size, that share a conserved tertiary structure composed of a flexible N-terminus, an N-loop, a three-stranded antiparallel -sheet, and a C-terminal -helix. Based on the arrangement of the conserved N-terminal cysteine residues, they are classified into the CXC, CC, XC, and CX3C subfamilies [16, 17]. Although initially conceptualized primarily as soluble chemoattractants, chemokines are now understood to function within highly structured tissue contexts in which matrix binding, oligomerization, receptor pharmacology, ligand scavenging, and local production–consumption dynamics collectively determine the geometry and biological meaning of chemotactic fields [16, 17]. In tumors, these gradients do not merely influence whether leukocytes enter or fail to enter the lesion; they also govern where distinct myeloid and lymphoid populations accumulate, how long they are retained, and whether their positioning supports cytotoxic immunity, stromal exclusion, vascular remodeling, or immunoregulatory dominance. Accordingly, the chemokine system in cancer is best viewed not as a set of isolated ligand–receptor pairs, but as an integrated spatial signaling framework in which gradient shape, receptor hierarchy, and tissue compartmentalization jointly define cellular behavior [16, 17]. This view is particularly relevant in the TME, where chronic inflammation, hypoxia, extracellular matrix remodeling, aberrant vasculature, and persistent cytokine exposure alter both chemokine availability and receptor responsiveness [16–18]. Understanding how these variables are integrated at the molecular and cellular levels is therefore essential for explaining immune topography in tumors and for rationally manipulating leukocyte trafficking in cancer therapy [16, 17]. The mechanisms described in this section—including glycosaminoglycan-mediated chemokine presentation, oligomerization, receptor activation, signal desensitization, and atypical chemokine receptor–mediated gradient editing—are established features of chemokine biology derived primarily from biochemical, cellular, and experimental tissue studies. Their relevance to exercise-associated remodeling of the intratumoral chemokine landscape should therefore be regarded as a mechanistic framework rather than as direct evidence that exercise induces these changes in human tumors. Given the intricate nature of these molecular interactions and the possibility that exercise may modulate some components of this system, the core chemokine axes potentially relevant to TME remodeling are synthesized in Supplementary Table S3. This table summarizes the molecular syntax, spatial dynamics, and potential mechanistic relevance of the CXCL9/10/11–CXCR3, CCL5–CCR5, and CXCL12–CXCR4 axes, with particular emphasis on how physiological exercise protocols could influence these pathways and on the distinction between established chemokine biology and proposed exercise-mediated effects.

### Spatial organization of chemokines: from diffusion to directionality

Directed cell migration requires more than ligand abundance; it requires spatial asymmetry that can be sensed, interpreted, and translated into stable front–rear polarity [18]. In tissues, this is achieved by converting freely diffusible chemokines into structured gradients whose steepness, persistence, and anatomical confinement are shaped by the biochemical properties of the ligand and by the physical characteristics of the surrounding matrix [11]. Within the TME, this process is especially important because interstitial fluid flow, elevated tissue pressure, matrix densification, and vascular leakiness can dissipate soluble signals and reduce directional fidelity [19]. A central determinant of chemokine localization is binding to glycosaminoglycans (GAGs), particularly heparan sulfate and related sulfated polysaccharides present on endothelial surfaces, stromal cells, and extracellular matrix components. Chemokine–GAG interactions are mediated primarily through electrostatic attraction between negatively charged sulfate and carboxyl groups on GAG chains and basic amino acid clusters, often enriched in lysine and arginine residues, within exposed regions of the chemokine scaffold [20]. These interactions restrict diffusion, protect ligands from convective loss, and promote their presentation in a tissue-bound form. As a result, migrating cells frequently respond not to a purely soluble gradient but to a composite field that includes immobilized, matrix-associated chemokine cues [20]. This distinction is biologically important because immobilized chemokines support haptotactic guidance and can generate sharper positional information than would be achieved by unrestricted diffusion alone. GAG binding also influences chemokine oligomerization, and this has important consequences for gradient architecture and receptor activation. Many chemokines exist in monomer–dimer equilibria, and some are capable of forming higher-order oligomers under conditions of elevated local concentration or matrix association [20]. In vivo, oligomerization has been reported to enhance tissue retention in selected chemokine systems and may facilitate the formation of high-avidity chemokine depots on endothelial or stromal surfaces. In some settings, oligomeric presentation is required for efficient leukocyte recruitment, particularly when stable luminal display is needed to support integrin activation and transendothelial migration [8–11, 16–21]. At the same time, oligomerization is not functionally neutral: changes in quaternary structure can alter receptor engagement, signaling bias, and migratory output. For selected chemokines, increased local concentration and dimer stabilization have been associated with reduced chemotactic efficiency or with signaling states that favor cell arrest rather than persistent directional migration. These effects may be particularly relevant in tumors, where confined production, matrix sequestration, and stromal crowding can generate microdomains of high local ligand concentration [8–11, 16–21]. The capacity of a chemokine gradient to instruct movement ultimately depends on how it is decoded by chemokine receptors, which belong predominantly to the class A rhodopsin-like family of seven-transmembrane G protein-coupled receptors (GPCRs). Structural and functional studies support a two-step binding mechanism in which receptor recognition begins with interaction between the receptor N-terminus and extracellular loops and the globular core of the chemokine, followed by insertion of the chemokine N-terminus into the receptor transmembrane bundle [8–11, 16–21]. The first step confers much of the binding specificity and affinity, whereas the second drives the conformational rearrangements required for receptor activation. These rearrangements alter the architecture of the intracellular receptor face, enabling coupling to heterotrimeric G proteins, most commonly of the Gαi family.

Following activation, GDP–GTP exchange on Gαi promotes dissociation of Gαi from the Gβγ complex, and both arms contribute to migratory signaling [11, 16, 17, 19, 21]. Gαi-dependent inhibition of adenylyl cyclase lowers intracellular cAMP, whereas Gβγ activates effectors such as phospholipase C- and phosphoinositide 3-kinase [11, 16, 17, 19, 21]. Hydrolysis of phosphatidylinositol 4,5-bisphosphate generates inositol 1,4,5-trisphosphate and diacylglycerol, supporting rapid calcium mobilization and downstream kinase activation. In parallel, localized production of phosphatidylinositol 3,4,5-trisphosphate at the leading edge recruits guanine nucleotide exchange factors and other polarity regulators that activate Rac1 and Cdc42, thereby promoting actin polymerization, lamellipodial extension, and directional sensing [11, 16, 17, 19, 21]. RhoA and associated contractile machinery contribute to uropod retraction and rear-end tension, stabilizing front–rear asymmetry [11, 16, 17, 19, 21]. The net result is not simply receptor occupancy, but spatially restricted signaling that converts extracellular ligand differences into polarized cytoskeletal remodeling. Importantly, receptor activation is not a binary event, and the same receptor can support distinct biological outputs depending on ligand identity, ligand presentation state, receptor density, and the recent signaling history of the cell [11, 16, 17, 19, 21]. This is particularly evident in biased signaling, whereby different ligands stabilize distinct receptor conformations and preferentially engage partially divergent downstream pathways [11, 16, 17, 19, 21]. The CCR7 ligands CCL19 and CCL21 provide a well-established example. Both ligands activate CCR7 and can promote chemotaxis, yet they differ in their capacity to induce receptor phosphorylation, -arrestinre cruitment, internalization kinetics, and signal persistence [11, 16, 17, 19, 21]. These differences are not merely biochemical curiosities; they influence how dendritic cells and T cells interpret tissue and lymphatic guidance cues, especially in environments where ligand distribution is spatially segregated between stromal surfaces, lymphatic endothelium, and draining lymphoid structures. In tumors, comparable ligand-dependent differences in receptor output may shape whether immune cells sustain migration, pause for antigen sampling, or become retained within stromal niches [11, 16, 17, 19, 21]. The fidelity of gradient sensing also depends on desensitization and receptor prioritization. Persistent receptor stimulation would otherwise saturate signaling pathways and collapse directional responsiveness. Homologous desensitization occurs when agonist-engaged receptors are phosphorylated by G protein-coupled receptor kinases, creating docking sites for -arrestinre that sterically limit further G-protein coupling and promote receptor internalization through clathrin-dependent pathways. Internalized receptors may subsequently recycle back to the plasma membrane or be targeted for degradation, depending on receptor type and cellular context [9, 11, 16, 17, 19–21]. Heterologous desensitization adds a second layer of regulation, as kinases activated downstream of other receptors, including protein kinase A and protein kinase C, can phosphorylate chemokine receptors independently of direct ligand engagement [9, 11, 16, 17, 19–21]. Through this mechanism, cells exposed to multiple inflammatory signals can impose a functional hierarchy among competing chemotactic inputs. Such prioritization is highly relevant in the TME, where tumor-derived chemokines, inflammatory mediators, growth factors, and danger signals coexist and frequently compete for control over leukocyte positioning. In addition to signal attenuation within responding cells, chemokine gradients are shaped extracellularly by atypical chemokine receptors (ACKRs), which function primarily as ligand scavengers, transporters, or gradient modulators rather than as classical chemotactic receptors [9, 11, 16, 17, 19–21]. ACKRs share the seven-transmembrane architecture of conventional chemokine receptors but lack the canonical signaling properties required for productive G -protein-driven migration. Instead, they regulate chemokine availability by constitutive or ligand-induced internalization, intracellular trafficking, transcytosis in selected settings, and ligand sequestration or degradation [9, 11, 16, 17, 19–21]. ACKR3, for example, binds CXCL12 with high affinity and can markedly limit local ligand accumulation, thereby preserving or reshaping CXCR4-directed gradients. ACKR4 similarly controls the tissue distribution of CCL19 and CCL21 and thereby influences CCR7-dependent cell trafficking [9, 11, 16, 17, 19–21]. These receptors are especially important in spatially restricted tissues because they establish sink functions that sharpen ligand fields, prevent excessive receptor saturation, and maintain directional contrast across short anatomical distances [9, 11, 16, 17, 19–21]. Taken together, gradient formation in the TME emerges from the interaction of at least four coupled processes: localized chemokine production, matrix tethering and restricted diffusion, receptor-mediated interpretation by migrating cells, and active gradient editing by scavenging systems. None of these processes operates in isolation [9, 11, 16, 17, 19–21]. A chemokine that is abundantly produced but poorly retained may fail to create stable positional information; a matrix-bound ligand that strongly accumulates may enhance recruitment in one region while promoting arrest or retention in another; and a receptor that is highly expressed may nevertheless become functionally subordinate if desensitized by competing inflammatory pathways [9, 11, 16, 17, 19–21]. The biological meaning of a chemokine gradient in cancer therefore resides not only in its composition, but in its topology, persistence, presentation state, and the signaling competence of the responding cell population. These principles become particularly evident when chemokine fields are examined at the level of tumor microanatomy. Rather than producing uniform leukocyte infiltration, tumors generate spatially discrete niches in which distinct chemokine programs recruit and retain different immune lineages [22]. Such compartmentalization is central to the emergence of immune-inflamed, immune-excluded, and immune-desert phenotypes, and it also underlies the segregation of macrophage, dendritic cell, and lymphocyte subsets into functionally divergent territories within the same lesion [9, 11, 16, 17, 19–21].

At the tissue level, chemokine gradients in tumors are organized into anatomically restricted niches that impose lineage-specific patterns of recruitment and retention [23]. This is particularly evident within the myeloid compartment, where spatial transcriptomics and single-cell profiling have shown that macrophage organization is better explained by ontogeny, anatomical localization, and chemokine specialization than by the conventional M1/M2 dichotomy [23, 24]. In several tumor settings, including lung cancer, tissue-resident interstitial macrophages and monocyte-derived recruited macrophages occupy distinct microenvironments and contribute differently to local immune architecture. Resident interstitial macrophage populations can be subdivided into transcriptionally and functionally distinct states, including CD206 ^hi^ and CD206 ^lo^ subsets [11, 19, 21, 24]. CD206 ^hi^ populations, often enriched for markers such as Folr2 and Cd163, are preferentially associated with bronchovascular or perivascular regions and may contribute to chemokine milieus enriched in CXCL9, CXCL10, and CXCL13. Through these ligands, resident myeloid cells may support the recruitment and spatial organization of CXCR3-expressing effector T cells and B cells, potentially contributing to the development or maintenance of tertiary lymphoid structures (11, 19, 21, 24). In this context, chemokine gradients do not simply increase immune cell density; they generate organized immune microdomains that support antigen presentation, lymphocyte interaction, and more effective local immune surveillance [24]. By contrast, monocyte-derived macrophages are often recruited through CCL2–CCR2-dependent pathways into tumor-core regions characterized by hypoxia, matrix remodeling, and inflammatory stress [9–11, 16, 20, 21, 24]. Within these compartments, infiltrating Ly6C⁺CCR2⁺ monocytes can give rise to macrophage states that have been associated with extracellular-matrix remodeling, suppressive cytokine production, and, in some settings, regulatory T-cell accumulation. The importance of this axis lies not only in monocyte recruitment per se, but in the establishment of a self-reinforcing spatial program in which tumor-associated chemokines, stromal remodeling, and myeloid differentiation converge to generate intratumoral suppressive niches [9–11, 16, 20, 21, 24]. Thus, the same broad chemokine system can support either immune organization or immune exclusion depending on where ligands are produced, how they are retained, and which responding populations dominate the local receptor landscape. A related principle applies to monocyte-derived dendritic cells, whose trafficking behavior may diverge from that of conventional dendritic cell subsets. In some tumor contexts, monocyte-derived dendritic cells migrate through chemokine axes that favor their accumulation in draining lymph nodes or immunoregulatory microenvironments, where they can attenuate rather than enhance effective T-cell priming [9–11, 16, 20, 21, 24]. Experimental disruption of selected pathways has suggested that interfering with suppressive myeloid-cell trafficking may improve the balance between tolerogenic and immunostimulatory antigen presentation, particularly when combined with vaccination or checkpoint-based strategies. These observations underscore that chemokine-directed migration cannot be interpreted solely in terms of total leukocyte influx; the identity, differentiation state, and spatial destination of recruited cells are equally important determinants of outcome [8–11, 16, 18, 20–24]. At the level of whole-tumor immune architecture, these spatial programs contribute to the emergence of three broadly recognized phenotypes. Immune-inflamed tumors are characterized by intratumoral accumulation of cytotoxic lymphocytes and are commonly associated with IFN- inducible chemokines such as CXCL9, CXCL10, and CXCL11, often accompanied by CXCL16-mediated recruitment or retention of CXCR6-expressing cells. In such lesions, chemokine gradients are generally associated with the penetration of effector cells into the tumor parenchyma rather than their confinement to the periphery [8–11, 16, 18, 20–24]. Immune-excluded tumors, by contrast, may generate substantial chemokine activity at the invasive margin yet still fail to support deep T-cell entry because stromal barriers, fibroblast-derived factors, myeloid suppressor cell accumulation, and matrix-bound chemokine sequestration prevent effective transit into tumor nests [8–11, 16, 18, 20–24]. CXCL12-rich stromal territories may be particularly relevant in this regard because they can generate retention zones that spatially confine lymphocytes outside regions of productive tumor-cell contact. Immune-desert tumors represent a more severe failure of immune organization, with limited recruitment of effector lymphocytes, poor cDC1 accumulation, and relative enrichment of regulatory or wound-healing programs, often accompanied by chemokine environments that favor suppressive populations over cytotoxic ones [8–11, 16, 18, 20–24]. Collectively, these observations support a model in which chemokine gradients in the TME function as spatial organizers of immune ecology rather than as simple chemoattractant cues. However, the extent to which exercise can reproduce or reconfigure these spatial programs within human tumors remains uncertain and requires direct experimental evaluation. Their effects depend on ligand biochemistry, matrix association, receptor signaling competence, scavenger receptor activity, and the anatomical logic of the tissue in which they are deployed. A mechanistic understanding of this system is essential not only for interpreting tumor immune phenotypes but also for designing interventions that reconfigure leukocyte positioning, promote productive lymphoid organization, and overcome stromal or myeloid barriers to antitumor immunity [8–11, 16, 18, 20–24]. However, the extent to which exercise can reproduce or reconfigure these spatial programs within human tumors remains uncertain and requires direct experimental evaluation. To systematically map these biophysical, biochemical, and spatial dynamics, the molecular mechanisms governing chemokine gradient formation, receptor signaling, and spatial compartmentalization within the TME are detailed in Supplementary Table S4. This synthesis connects the micro-level biophysical properties of chemokines (e.g., GAG tethering, oligomerization, and signaling bias) to tissue-level immune phenotypes and localized cellular destinations, while distinguishing established chemokine biology from proposed exercise-related mechanisms.

### The failure of homing in solid tumors: biochemical and physical barriers

The major biochemical, vascular, stromal, and mechanical mechanisms underlying immune-cell homing failure in solid tumors are summarized in Supplementary Table S5, with emphasis on their cellular level of action, molecular mediators, and potential relevance to exercise-associated microenvironmental remodeling. The mechanisms underlying homing failure are supported by evidence from experimental tumor models and, in some cases, human tumor studies; however, whether exercise can directly reverse these barriers within human tumors remains incompletely demonstrated. Accordingly, the exercise-related implications discussed in this section should be interpreted as biologically plausible mechanisms rather than established effects. The inability of effector immune cells to home efficiently into solid tumors is not simply a consequence of insufficient antigen recognition or poor systemic immune activation [25]. The barriers described below are well established in tumor immunology; however, their potential modulation by exercise should be interpreted as a mechanistic hypothesis rather than as direct evidence of exercise-induced reversal of immune exclusion in human tumors. Rather, it reflects a multilayered failure of trafficking that emerges from the combined action of biochemical suppression, endothelial dysfunction, stromal exclusion, and mechanical restraint [26]. In healthy inflammatory settings, leukocyte homing is coordinated by a highly ordered sequence of events that includes rolling, activation by chemokines displayed on the vascular surface, firm adhesion through integrins, transmigration across the endothelium, and subsequent navigation through tissue microdomains. Solid tumors, however, progressively distort each step of this program [26]. As a result, immune cells that are present in the circulation or in draining lymphoid tissues often fail to enter the lesion, fail to penetrate beyond the stromal boundary, or fail to persist once they reach the tumor bed [21, 26]. At the biochemical level, tumors frequently create chemokine landscapes that are quantitatively high but functionally ineffective [27, 28]. Rather than generating productive gradients that guide cytotoxic lymphocytes into tumor nests, malignant and stromal cells may produce chemokines in spatially restricted or mislocalized patterns that retain cells in perivascular or stromal compartments. In several tumor models, CXCL12 provides a well-characterized example of a chemokine axis that can contribute to barrier-forming or retention-associated signaling [27, 28]. Produced by cancer-associated fibroblasts, pericytes, and other stromal elements, CXCL12 can create a retention field that traps T cells and other CXCR4-expressing leukocytes at the invasive margin, thereby preventing deep parenchymal infiltration [8–11, 16–23, 25, 27, 28]. This stromal chemokine architecture is often reinforced by high-affinity interactions with glycosaminoglycans, which immobilize ligands and convert potentially migratory cues into spatially confining signals. In this setting, the issue is not the absence of chemokines, but the failure of those cues to generate a permissive migratory topology. Tumors also modify chemokine signaling through proteolytic editing. Chemokines can be cleaved, truncated, oxidatively modified, or otherwise altered by proteases released from tumor cells, neutrophils, macrophages, and stromal cells [8–11, 16–23, 25, 27, 28]. These modifications may reduce receptor affinity, eliminate directional activity, or shift the balance between attraction and arrest. Inflammatory proteases such as matrix metalloproteinases, cathepsins, and dipeptidyl peptidase IV-like enzymes can reshape the chemokine repertoire; for selected chemokines, this processing has been reported to reduce attractant activity or generate functionally altered, including antagonistic, species. Because chemokine function is highly dependent on N-terminal integrity, even subtle biochemical modifications can substantially impair receptor engagement and downstream signaling. In addition, atypical chemokine receptors can intensify this problem by scavenging ligands and lowering the local concentration of signals needed for stable homing [8–11, 16–23, 25, 27, 28]. Thus, in many tumor contexts, the biochemical environment can favor ligand depletion, ligand inactivation, or ligand mispresentation rather than productive recruitment [8–11, 16–23, 25, 27, 28].

A further biochemical layer of suppression arises from the inflammatory and metabolic state of the TME itself [8–11, 16–23, 25, 27, 28]. Hypoxia, acidosis, elevated lactate, adenosine accumulation, and reactive oxygen species collectively alter both chemokine production and leukocyte responsiveness [8–11, 16–23, 25, 27, 28]. Hypoxia-inducible pathways can induce chemokine expression in some compartments while simultaneously promoting the accumulation of suppressive myeloid cells and vascular abnormalities that blunt cell entry [8–11, 16–23, 25, 27, 28]. Acidic extracellular pH and lactate-rich conditions have been shown in experimental settings to reduce T-cell motility, impair cytoskeletal dynamics, and weaken chemokine-driven migration. Adenosine, acting through A2A receptor signaling, further dampens T-cell activation and migratory competence, creating a tissue environment in which even properly formed chemokine gradients may fail to elicit productive infiltration. In this sense, biochemical homing failure is not simply a matter of ligand availability, but of signal interpretation within a metabolically hostile milieu [8–11, 16–23, 25, 27, 28]. The vascular barrier is equally important. Tumor blood vessels are typically abnormal in structure and function, with irregular branching, poor pericyte coverage, heterogeneous permeability, and unstable endothelial phenotypes [8–11, 16–23, 25, 27, 28]. These abnormalities compromise the expression and spatial organization of adhesion molecules and alter the endothelial presentation of chemokines required for leukocyte arrest and transendothelial migration. In physiological homing, chemokines displayed on endothelial surfaces activate leukocyte integrins such as LFA-1 and VLA-4, enabling firm adhesion to ICAM-1 and VCAM-1. In tumors, however, endothelial cells may express these molecules at suboptimal levels or in a disorganized manner, reducing the efficiency of rolling-to-arrest transitions [8–11, 16–23, 25, 27, 28]. Tumor endothelial cells can also adopt an anergic or anti-inflammatory phenotype under the influence of VEGF, endothelin-1, prostaglandins, and other suppressive mediators, thereby limiting their ability to support chemokine presentation and adhesion-dependent recruitment. VEGF-driven angiogenesis is particularly relevant because it promotes vessels that are structurally immature and functionally non-permissive. Such vessels are frequently compressed by surrounding tumor mass and stromal cells, which reduces perfusion and creates irregular flow patterns [8–11, 16–23, 25, 27, 28]. The consequence is a paradoxical state in which the tumor may be vascularized but still poorly accessible to immune cells. Reduced perfusion limits both leukocyte delivery and the establishment of stable endothelial activation states. In some experimental settings, vascular-normalizing interventions have been associated with improved immune-cell trafficking, potentially through improved vessel architecture, endothelial adhesion-molecule expression, and accessibility of chemokines presented at the endothelial interface. This observation underscores the principle that immune homing failure is often a vascular as much as a biochemical problem. Beyond the endothelium, the extracellular matrix imposes a formidable physical barrier. Solid tumors are commonly enriched for fibrillar collagens, fibronectin, hyaluronan, and cross-linked matrix networks that increase tissue stiffness and narrow interstitial spaces [8–11, 16–23, 25, 27, 28]. These changes elevate interstitial fluid pressure, compress microvessels, and hinder leukocyte movement. T cells and other lymphocytes are highly deformable but still require sufficient space, matrix permissiveness, and coordinated proteolysis to migrate effectively through dense stromal regions. In desmoplastic tumors, the matrix can function as a mechanical sieve that excludes cells from tumor nests even when they have successfully extravasated from the vasculature. Cancer-associated fibroblasts are central to this process, since they not only produce matrix components but also organize collagen alignment, generate contractile tension, and secrete chemokines and growth factors that reinforce spatial segregation [8–11, 16–23, 25, 27, 28].

Transforming growth factor-β is one of the major mediators linking stromal fibrosis to immune exclusion. By promoting fibroblast activation, extracellular matrix deposition, and vessel compression, TGF-β contributes to a tissue architecture in which effector cells are physically and functionally confined to the tumor periphery [29]. TGF-β also dampens the transcriptional programs required for cytotoxic T-cell differentiation and may reduce the expression of receptors and adhesion molecules needed for tissue infiltration. Similar effects are observed with other stromal and myeloid suppressive signals that promote matrix remodeling while limiting productive immune access [29]. The result is a self-reinforcing exclusion zone in which the stromal boundary becomes an anatomical and biochemical checkpoint that circulating lymphocytes fail to cross [29] Myeloid cells further contribute to homing failure through the formation of suppressive vascular and stromal niches. Tumor-associated macrophages, neutrophils, and myeloid-derived suppressor cells can secrete VEGF, CXCL8, CCL2, IL-10, and matrix-remodeling enzymes that maintain aberrant vasculature and sustain an immunosuppressive extracellular environment [16, 18, 23, 25, 29]. In addition, these cells can consume local nutrients, generate reactive species, and interfere with T-cell motility and receptor signaling. In many tumors, the chemokine system is therefore not absent but diverted: signals that would ordinarily support infiltration are repurposed to recruit suppressive populations or to reinforce structural exclusion [16, 18, 23, 25, 29].

Importantly, these barriers are not independent. Biochemical, vascular, and mechanical constraints are tightly coupled and often mutually amplifying [16, 18, 23, 25, 29]. A fibrotic matrix compresses vessels; compressed vessels worsen hypoxia; hypoxia promotes vascular dysfunction and suppressive myeloid recruitment; suppressive myeloid cells further remodel matrix and degrade effective chemokine gradients. This coupled system explains why the failure of homing in solid tumors is so resistant to simple chemokine supplementation [16, 18, 23, 25, 29]. Introducing an attractant alone is rarely sufficient when the tissue environment has been remodeled to prevent ligand presentation, endothelial adhesion, or physical entry. Effective restoration of homing is therefore likely to require simultaneous manipulation of chemokine gradients, endothelial permissiveness, stromal mechanics, and local immunometabolism [16, 18, 23, 25, 29].

Viewed in this way, the failure of immune cell homing in solid tumors is best understood as a failure of tissue organization. The tumor does not merely repel immune cells; it actively reprograms the microenvironment so that recruitment cues become spatially misdirected, endothelial gateways become unresponsive, and stromal architecture becomes exclusionary [16, 18, 23, 25, 29]. This integrated barrier system is a major reason why many solid tumors remain inaccessible to effector lymphocytes despite the presence of recognizable antigens and systemic immune activation. Therapeutic strategies aimed at reversing immune exclusion will therefore need to restore not only immune activation, but also the biochemical and physical conditions required for homing to occur [16, 18, 23, 25, 29] The major barriers underlying immune-cell exclusion are summarized in Table 1.(Table).

**Table 1:** Extended mechanistic framework of chemokine gradient syntax and homing failure in the tumor microenvironment

Mechanistic layer	Molecular architecture / key mechanism	Principal molecules	Functional output in the TME	Translational implication
Ligand immobilization on tissue scaffolds	Chemokines are not distributed as freely diffusible cues; many are tethered to glycosaminoglycans on endothelial and stromal surfaces, which constrains diffusion and creates spatially fixed signaling fields	Heparan sulfate, chondroitin sulfate, proteoglycans, CXCL12, CCL19, CCL21, CXCL9, CXCL10, CXCL13	Generates localized chemokine hotspots, preserves directional information, and enables cell tracking along matrix-bound gradients rather than bulk concentration gradients	GAG-targeting strategies, stromal remodeling, or engineered chemokine variants may alter gradient topology and immune cell positioning
Chemokine oligomerization and avidity control	Oligomerization increases local retention, multivalent receptor engagement, and effective ligand density; it also modifies protease susceptibility and matrix binding	CXCL12, CCL19, CCL21, CCL2, CXCL8, heparan sulfate-binding motifs	Enhances gradient stability and supports sustained leukocyte arrest or retention in defined niches	Ligand engineering or oligomerization-disrupting interventions could reshape chemokine persistence and bioavailability
Two-site GPCR recognition and receptor triggering	Canonical chemokine receptors recognize ligands through a two-step model: initial docking via receptor N-terminus and subsequent insertion of the chemokine N-terminus into the receptor binding pocket, enabling activation	CXCR4, CCR7, CXCR3, CCR2, CCR5, GPCR extracellular loops, receptor N-terminus	Converts spatial ligand information into directed intracellular signaling and migratory polarity	Selective receptor antagonists, biased agonists, or allosteric modulators can reprogram receptor output rather than simply block binding
G protein-dependent migration signaling	Receptor activation typically engages G^i^ proteins, releasing Gβγ subunits to activate PI3K, PLCβ, and small GTPases; this induces actin polymerization, protrusion formation, and chemotaxis	CXCR4, CCR7, CXCR3, CCR2, CCR5, GPCR extracellular loops, receptor N-terminus	Converts spatial ligand information into directed intracellular signaling and migratory polarity	Selective receptor antagonists, biased agonists, or allosteric modulators can reprogram receptor output rather than simply block binding
Biased signaling and pathway selectivity	The same receptor can preferentially route signaling through G protein- or β-arrestin-dominant outputs, generating distinct migratory or desensitization phenotypes depending on ligand context	*β*-arrestins, CXCR4, CCR7, ligand variants, receptor phosphorylation machinery	Determines whether signaling promotes migration, retention, arrest, or receptor internalization	Biased ligands may allow selective enhancement of immune recruitment while limiting maladaptive signaling
Desensitization, internalization, and signal termination	Sustained stimulation triggers receptor phosphorylation by GRKs, β-arrestin recruitment, and endocytosis, thereby limiting chronic signaling and enabling receptor recycling or degradation	GRKs, *β*-arrestin, CXCR4, CCR7, CCR5	Prevents overstimulation but can blunt sustained chemotactic responsiveness in chronically inflamed or tumor-exposed cells	Interventions that preserve receptor responsiveness may improve cell migration into chemokine-rich tumor regions
ACKR-mediated gradient editing	Atypical chemokine receptors scavenge, transcytose, or redistribute chemokines without canonical G protein signaling, thereby sculpting ligand availability and sharpening or erasing gradients	ACKR1, ACKR2, ACKR3, ACKR4, CXCL12, CCL19, CCL21, inflammatory CC chemokines	Controls gradient amplitude, spatial spread, and sink-source behavior; can either support or suppress immune positioning depending on context	ACKR targeting offers a route to reprogram chemokine fields rather than merely augment ligand levels
Proteolytic editing of chemokines	Chemokines may be truncated by proteases, altering receptor selectivity, potency, or scavenging efficiency; some truncations convert a chemoattractant into a weak agonist or antagonist	DPP4/CD26, MMPs, cathepsins, CXCL12, CCL2, CXCL9/10/11	Reduces gradient integrity and can generate nonproductive or misdirecting chemokine signals	Protease inhibition may restore functional chemokine gradients and improve immune trafficking
Chemokine clustering within the stromal niche	Chemokines are organized into microdomains influenced by ECM density, fibroblast activity, and vascular architecture, creating discrete routes for leukocyte movement	CAFs, fibroblasts, collagen, fibronectin, hyaluronan, CXCL12, CCL2	Produces compartmentalized recruitment patterns, often favoring perivascular retention or stromal sequestration over parenchymal penetration	Stromal decompaction and CAF reprogramming may open migration corridors for effector cells

**Table 1:** **continued**: Failure of homing in solid tumors: biochemical, vascular, and physical barriers

Mechanistic layer	Molecular architecture / key mechanism	Principal molecules	Functional output in the TME	Translational implication
Endothelial anergy	Tumor endothelium often fails to upregulate adhesion and transmigration programs in response to inflammatory cues, rendering vessels non-permissive to immune-cell entry	VEGF, endothelin, prostaglandins, ICAM-1, VCAM-1, E-selectin, P-selectin	Impairs rolling, firm adhesion, and transendothelial migration, producing vascular immune exclusion	Vascular normalization and endothelial reactivation can restore leukocyte access
Defective rolling and capture	Initial leukocyte tethering depends on selectin-mediated interactions; when endothelial selectins are low or dysfunctional, leukocytes fail to slow sufficiently for subsequent integrin activation	E-selectin, P-selectin, PSGL-1, CD44, sLe* x*	Reduces leukocyte capture from blood flow and limits the initiation of the homing cascade	Selectin restoration or endothelial activation may improve first-contact recruitment
Impaired integrin activation	Chemokine-triggered inside-out signaling fails when receptor signaling is weak, desensitized, or spatially uncoupled from endothelial ligands, preventing transition to high-affinity integrin states	CXCR3, CXCR4, CCR2, LFA-1, VLA-4, talin, kindlin, Rap1	Weakens firm adhesion to ICAM-1/VCAM-1 and blocks stable arrest on the vessel wall	Therapies that restore chemokine responsiveness may improve arrest and extravasation
Adhesion molecule suppression on endothelium	Tumor-derived factors downregulate endothelial adhesion ligands and blunt inflammatory activation, reducing the molecular platform needed for transmigration	VEGF, TGF-β, IL-10, ICAM-1, VCAM-1, JAMs, PECAM-1	Prevents leukocyte arrest and diapedesis even in the presence of systemic inflammation	Endothelial reprogramming may be more effective than simply increasing lymphocyte numbers
Chemokine mispresentation at the vascular interface	Chemokines may be produced but not properly displayed on endothelial surfaces, or may be retained in nonproductive stromal compartments away from the vasculature	CXCL12, CCL2, CCL5, heparan sulfate, proteoglycans	Creates a misleading chemokine landscape that fails to guide cells across the vessel wall and instead traps them in perivascular zones	Repositioning chemokines toward the vessel interface may restore directional entry
CXCL12-driven stromal barricades	CAF-derived CXCL12 can form peritumoral or perivascular barriers that retain T cells in stromal regions and prevent deep parenchymal infiltration	CXCL12, CXCR4, CXCR7/ACKR3, CAFs, collagen-rich stroma	Promotes immune exclusion and spatial segregation of effector cells from tumor nests	CXCL12/CXCR4-axis inhibition may convert excluded tumors into infiltrated phenotypes
Proteolytic erosion of chemokine function	Proteases in the TME degrade chemokines or truncate their N-termini, diminishing receptor affinity and directional precision	MMPs, DPP4/CD26, cathepsins, CXCL9, CXCL10, CXCL12	Produces weak, truncated, or antagonist-like ligands that cannot sustain productive chemotaxis	Protease blockade may preserve chemokine bioactivity and infiltration signals
Hypoxia-dependent trafficking suppression	Hypoxia alters endothelial phenotype, metabolic state, and chemokine receptor signaling, while promoting immunosuppressive transcriptional programs	HIF-1α, HIF-2α, adenosine axis, VEGF, CXCL12	Limits vascular accessibility, reduces T cell motility, and supports suppressive myeloid recruitment	Hypoxia-pathway targeting may improve vascular permissiveness and immune entry
Acidosis and lactate-mediated motility defects	Acidic and lactate-rich environments impair actin remodeling, receptor signaling competence, and effector cell persistence	Lactate, MCT1/MCT4, pH stress, Rho GTPases, actin regulators	Reduces T-cell motility, migration persistence, and killing capacity once cells enter the tumor	Metabolic buffering or lactate-axis modulation may restore migratory function
Fibrotic matrix compression and interstitial resistance	Dense collagen, crosslinked ECM, and elevated interstitial pressure physically impede immune-cell movement and compress vessels	Collagen I/III, LOX, fibronectin, hyaluronan, TGF-β, CAFs, interstitial fluid pressure	Restricts both vascular access and interstitial penetration, promoting immune-excluded architecture	Stromal softening, LOX inhibition, and CAF reprogramming may improve penetration
CAF-driven immune compartmentalization	CAF subsets generate chemokine-rich but functionally restrictive niches that sequester cells rather than guiding them into tumor nests	CAFs, CXCL12, CCL2, TGF-β, IL-6, FAP	Supports peritumoral accumulation, myeloid recruitment, and exclusion of cytotoxic lymphocytes	CAF targeting must be nuanced; broad ablation may differ from reprogramming strategies
Failure of transendothelial migration	Even when adhesion occurs, abnormalities in endothelial junctional remodeling and cytoskeletal coordination can block diapedesis	PECAM-1, JAM-A, VE-cadherin, ICAM-1, RhoA, Src, FAK	Cells arrest on the vessel wall but fail to cross into the tissue parenchyma	Enhancing junctional permissiveness may be required in addition to chemokine restoration
Immune-desert versus immune-excluded phenotypes	Distinct combinations of defective chemokine presentation, vascular dysfunction, and stromal barriers generate different non-inflamed states	CXCL9/10, CXCL12, VEGF, TGF-β, CAFs, endothelial adhesion molecules	Produces either a true lack of immune recruitment or a peripheral accumulation of cells that cannot penetrate tumor nests	Phenotype-specific interventions are needed; desert and excluded tumors are not mechanistically equivalent

## Exercise-mediated remodeling of the tumor chemokine landscape

Exercise should not be viewed simply as a systemic anti-inflammatory stimulus or as a nonspecific enhancer of immune surveillance. In the setting of solid tumors, its potential relevance lies in the possibility that it can alter the conditions that shape chemokine-guided immune-cell trafficking [30]. Leukocyte recruitment in tumors is often limited by defective endothelial activation, stromal exclusion, metabolic stress, and abnormal chemokine presentation. Against this background, exercise could influence immune access not only by changing the number of circulating effector cells, but also by modifying the vascular, stromal, and metabolic context in which chemokine signals are interpreted and acted upon. Whether these systemic and tissue-level effects translate into reproducible remodeling of chemokine-guided trafficking within human tumors remains uncertain [30–33]. The distinction between established biology and exercise-related inference is important in this context. The molecular and cellular mechanisms that regulate chemokine production, endothelial presentation, stromal retention, and receptor-dependent leukocyte trafficking are well established in experimental immunology and tumor biology. By contrast, direct evidence that exercise systematically remodels these processes within human tumors remains limited. Accordingly, the mechanisms discussed below should be interpreted as a framework for generating and testing exercise-related hypotheses, rather than as evidence that exercise universally normalizes the intratumoral chemokine landscape.

An increase in circulating CD8⁺ T cells or NK cells may have limited biological consequences if the tumor vasculature remains non-adhesive, if the stromal compartment continues to retain immune cells at the invasive margin, or if dominant chemokine programs favor myeloid recruitment and exclusion over cytotoxic infiltration [30–33]. From this perspective, exercise may be viewed as a regulator of chemokine ecology across several interconnected levels. At the systemic level, acute exercise mobilizes leukocyte subsets with immediate effector potential, redistributing NK cells, cytotoxic T lymphocytes, and other immune populations through catecholaminergic and hemodynamic mechanisms. Repeated exercise, however, appears to do more than induce transient mobilization [30–33]. Chronic exercise has been associated in experimental and systemic studies with changes in host inflammatory tone, myeloid-cell states, vascular function, and tissue oxygenation. These adaptations could, in principle, modify the balance between chemokine programs associated with immune activation and those linked to retention, suppression, or aberrant wound-healing responses; however, their occurrence and functional significance within human tumors remain incompletely defined [30–34]. Across selected preclinical models, exercise has been associated with increased intratumoral CD8⁺ T-cell accumulation, enhanced cytotoxic activity, and reductions in some suppressive immune populations. However, these findings are heterogeneous and do not establish that exercise directly remodels chemokine gradients or restores productive immune-cell homing in human tumors [30–35]. Mechanistically, the exercise-responsive chemokine landscape should be understood as the integrated output of at least four processes [30–35]. First, exercise can influence the transcriptional programs that determine which chemokines are produced, particularly those downstream of interferon signaling and inflammatory pattern recognition. Second, exercise can alter vascular and stromal architecture, thereby changing where chemokines are displayed, immobilized, or rendered functionally accessible at the endothelial interface and within the interstitium [30–35]. Third, exercise can shift the cellular composition of the tumor microenvironment, including macrophages, fibroblasts, endothelial cells, and lymphocyte subsets, each of which contributes differently to chemokine production, scavenging, or interpretation [36]. Fourth, exercise can modify the metabolic and oxygenation state of the tumor, thereby indirectly influencing chemokine expression through pathways linked to hypoxia-inducible signaling, lactate accumulation, and inflammatory resolution [37]. The implication is that exercise does not act on a single ligand–receptor pair in isolation; rather, it may recalibrate the hierarchy of chemokine cues that determine whether a tumor remains immune-deserted, immune-excluded, or progresses toward a more inflamed and infiltration-permissive state [30–35, 37]. This conceptual framework is especially important because not all chemokine changes induced by exercise should be interpreted as uniformly beneficial [30–35, 37]. A chemokine program that supports effector-cell entry in one context may also recruit suppressive myeloid cells, reinforce stromal retention, or sustain prometastatic inflammation in another [30–35, 37]. Accordingly, the effects of exercise on tumor chemokine biology are likely to be axis-specific, dose-sensitive, temporally dynamic, and strongly conditioned by tumor type, vascular phenotype, stromal density, and baseline immune contexture. The most defensible interpretation, therefore, is not that exercise universally “normalizes” tumor chemokine signaling, but that it may selectively favor the restoration of chemokine programs associated with cytotoxic recruitment while weakening those associated with exclusion, persistent inflammatory damage, or nonproductive retention. Within this framework, the most compelling evidence currently supports a role for exercise in reinforcing interferon-linked chemokine axes involved in effector T-cell recruitment, particularly the CXCL9/CXCL10/CXCL11–CXCR3 system, while more preliminary or context-dependent evidence pertains to the CCL5–CCR5 and CXCL12–CXCR4 pathways and to transcriptional or epigenetic mechanisms that may sustain these changes over time [30–35, 37]. The principal pathways through which exercise may remodel the tumor chemokine landscape are summarized in Supplementary Table S6, with emphasis on regulatory level, candidate chemokine axes, microenvironmental consequences, and the current strength of evidence.

### **Restoring** interferon-linked chemokine programs: **The CXCL9/10/11–CXCR3 Axis**

Among the chemokine programs implicated in productive antitumor immunity, the CXCL9/CXCL10/CXCL11–CXCR3 axis occupies a particularly important position because it links inflammatory sensing to directional effector-cell trafficking [38]. CXCR3 is prominently expressed on activated CD8^+^T cells, NK cells, and Th1-polarized lymphocytes, and its ligands—most notably CXCL9 and CXCL10, with context-dependent contributions from CXCL11—are typically induced in response to interferon-dominated immune states [38, 39]. In inflamed tumors, this axis functions not merely as a general chemoattractant system, but as a spatial routing mechanism that enables activated lymphocytes to recognize vascular exit sites, cross tumor endothelium, and accumulate within malignant tissue rather than remaining confined to perivascular or peripheral stromal regions [40]. The relevance of this pathway to tumor immunity is underscored by evidence showing that ligand availability for CXCR3 is closely associated with TIL-inflamed phenotypes, whereas reduced expression of CXCR3 ligands is linked to poor T-cell infiltration [38–40]. The mechanistic importance of this axis lies in its role at the vascular interface [40]. In tumor-bearing tissues, successful homing of cytotoxic lymphocytes requires more than the presence of effector cells in the bloodstream; it requires endothelial display of interpretable chemotactic signals capable of triggering integrin activation, firm adhesion, and transendothelial migration [41]. Evidence summarized in the melanoma trafficking literature indicates that CXCR3 engagement by CXCL9 and CXCL10 is functionally indispensable for T-cell passage across tumor-associated endothelium, suggesting that this axis acts at a critical checkpoint between circulating immune competence and actual intratumoral infiltration [41]. This has direct implications for exercise biology [40]. If exercise increases the number of mobilized effector cells but fails to restore chemokine-dependent endothelial guidance, then immune redistribution remains biologically incomplete. Conversely, if exercise enhances interferon-linked chemokine programs or improves the vascular conditions under which CXCR3 ligands are produced and presented, then it can convert transient systemic mobilization into meaningful tumor infiltration [42]. Current evidence supports this latter possibility, although the strength of evidence is not uniform across all mechanistic levels. Preclinical and mechanistic studies indicate that exercise can enhance intratumoral CD8^+^ T-cell accumulation and cytotoxic activity, and at least part of this effect has been linked to the CXCL9/11–CXCR3 pathway [20, 28, 42]. These observations are biologically plausible because exercise is capable of elevating IFN-, a central upstream driver of CXCL9, CXCL10, and CXCL11 expression in multiple stromal and immune compartments [20, 28, 42]. Once induced, these ligands can promote recruitment of CXCR3^+^ cytotoxic lymphocytes and NK cells, thereby amplifying local type 1 immunity [20, 28, 42]. In this sense, exercise may not simply add more immune cells to circulation; it may help restore the chemotactic syntax that allows inflamed tumors to become legible to those cells. The exercise-associated restoration of this axis is likely to operate through both direct and indirect mechanisms [20, 28, 42]. A direct mechanism would involve increased IFN- dependent induction of CXCR3 ligands by tumor-associated myeloid cells, endothelial cells, or stromal cells [20]. An indirect mechanism would involve remodeling of the tumor milieu such that CXCR3 ligand production becomes more sustainable or functionally useful. Exercise-induced improvements in perfusion and vascular architecture, together with attenuation of intratumoral hypoxia, may be particularly relevant here [20]. Hypoxia distorts endothelial responsiveness, sustains nonproductive inflammatory programs, and can reinforce recruitment patterns that favor suppressive myeloid populations over cytotoxic lymphocytes. By promoting a more physiologic vascular state and reducing HIF-1 linked dysfunction, exercise may increase the likelihood that interferon-inducible chemokines are not only produced but properly displayed within a trafficking-competent tissue context [20, 28, 42]. This distinction matters because chemokine abundance alone is insufficient; the ligands must be spatially available at sites where lymphocytes can decode them to execute endothelial arrest and tissue entry [38]. Another layer of relevance concerns the relationship between the CXCR3 axis and the broader architecture of immune exclusion. Tumors with impaired CXCL9/CXCL10/CXCL11 expression frequently exhibit a failure to recruit activated T cells despite the presence of antigenicity or systemic immune activation [43]. In such tumors, the defect is not necessarily the absence of immune cells altogether, but the absence of a chemokine program capable of converting immune recognition into tissue infiltration [43].

**Fig. 1:**
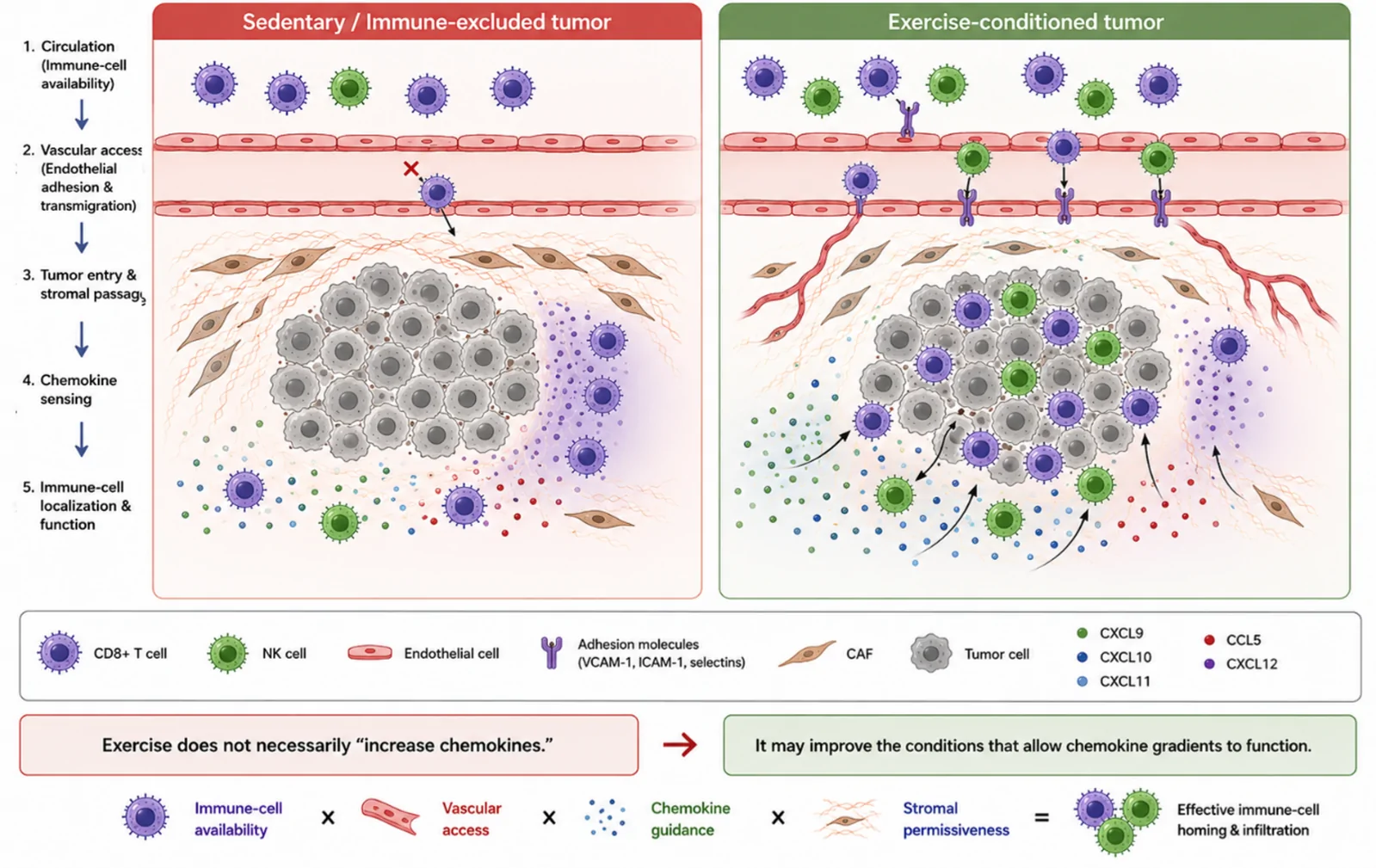
Exercise reconfigures the chemokine–vascular–stromal landscape governing immune-cell homing in tumors. Comparison of sedentary/immune-excluded and exercise-conditioned tumor states. Exercise may improve immune-cell availability, vascular access, stromal permissiveness, and chemokine-guided trafficking, thereby facilitating CD8⁺ T-cell and NK-cell localization within tumors. The model emphasizes that exercise may enhance the conditions that allow chemokine gradients to function rather than simply increasing chemokine levels. Created with BioRender.com

Exercise may partially counteract this defect by shifting the balance of cytokine and chemokine signaling away from a suppressive or wound-repair-dominant state and toward a type 1 inflammatory program [44]. The result, when successful, would be expected to include greater accumulation of CXCR3 CD8^+^T cells within the tumor parenchyma, enhanced granzyme-dependent cytotoxicity, and a transition from poorly infiltrated or excluded immune architectures toward a more inflamed phenotype [44]. However, this section must be interpreted with appropriate caution. The strongest available evidence currently supports the importance of the CXCL9/CXCL10/CXCL11–CXCR3 axis for T-cell trafficking in tumors and the capacity of exercise to augment antitumor immune infiltration, but direct human evidence demonstrating robust, exercise-induced upregulation of intratumoral CXCL9, CXCL10, or CXCL11 remains limited in the materials reviewed [44–46]. Therefore, a rigorous interpretation is that exercise plausibly restores interferon-linked chemokine programs that favor CXCR3-dependent trafficking, rather than that this restoration has been comprehensively demonstrated across tumor types and clinical settings [44–46]. This distinction is not semantic; it reflects a genuine evidentiary boundary. Still, even within that boundary, the axis remains one of the most biologically coherent pathways through which exercise could translate systemic immune mobilization into spatially effective tumor immunity [44–46]. From a translational standpoint, this has substantial implications. If exercise preferentially reinforces interferon-linked chemokine circuits, then it may function as a physiological adjunct that enhances the probability of effector-cell entry into tumors already capable of partial immune recognition but deficient in trafficking efficiency. Such an effect would be especially relevant in combination settings, including immune checkpoint blockade, adoptive cell therapy, or vascular-normalizing interventions, where the presence of activated T cells is not the limiting factor, but their localization within tumor tissue is. In that context, the CXCL9/CXCL10/CXCL11–CXCR3 axis may represent one of the most credible mechanistic bridges between exercise physiology and tumor immunology: a pathway through which systemic exertion becomes translated into improved chemotactic legibility of the tumor microenvironment for cytotoxic lymphocytes [44–46] Fig. 1. Key molecular, cellular, and microenvironmental features of the CXCL9/CXCL10/CXCL11–CXCR3 axis, including its relevance to exercise-mediated immune trafficking, are summarized in Supplementary Table S7.

### Exercise and context-dependent remodeling of the CCL5–CCR5 pathway

The CCL5–CCR5 axis occupies a uniquely ambivalent position within tumor immunobiology because it can support either antitumor leukocyte recruitment or tumor-promoting inflammatory circuitry depending on the cellular context, spatial compartment, and dominant immune ecology of the tumor microenvironment [47]. In contrast to chemokine systems that are more narrowly associated with effector-cell trafficking, CCL5 can function as a bidirectional organizer of immune cell positioning: on the one hand, it promotes the recruitment and intratumoral localization of CCR5-expressing CD8^+^T cells, NK cells, and Th1-polarized lymphocytes; on the other hand, it can attract CCR5 + myeloid populations such as MDSCs and tumor-associated macrophages, thereby reinforcing immune suppression, tissue remodeling, and in some settings even tumor progression [48]. The biology of CCL5–CCR5-mediated leukocyte recruitment is well established in inflammatory and tumor-immunology settings, but its modulation by exercise remains less clearly defined. In particular, direct evidence that exercise alters intratumoral CCL5 production, spatial presentation, CCR5 expression, or the functional distribution of CCR5-expressing cell populations in human tumors is limited. The exercise-related mechanisms discussed below should therefore be interpreted as context-dependent and hypothesis-generating unless supported by direct intratumoral evidence.

This duality is not a conceptual nuisance but the central biological feature of the axis. Any review that treats CCL5 as uniformly beneficial would be mechanistically incomplete [48]. At the level of cell trafficking, CCL5 is classically recognized as a chemotactic ligand for CCR5-expressing effector lymphocytes that have exited lymphoid tissues and are capable of tissue homing [49]. Within inflamed tumors, this ligand–receptor pair contributes to the directional migration of activated T cells and NK cells toward malignant tissue, where CCR5 engagement supports cytoskeletal polarization, integrin activation, and transmigration through the endothelial barrier [49]. Studies of tumor immunology have shown that T-cell-inflamed melanomas can exhibit a coordinated inflammatory chemokine landscape enriched in CCL5, alongside CCL2, CCL3, CCL4, CXCL9, CXCL10, CXCL11, and CXCL13, whereas poorly infiltrated tumors often show lower expression of, or less coordinated patterns involving, these chemotactic cues [38, 39, 42, 48]. In this context, CCL5 is not an isolated ligand but part of a broader “inflamed” chemokine syntax that permits immune entry into tumors rather than passive circulation around them [50]. Moreover, CCR5 expression is characteristic of expanded tumor-infiltrating lymphocytes, and CCR5–CCL5 expression correlates with increased T-cell infiltration and improved responsiveness to adoptive cell therapy [50]. These observations support the idea that CCL5 can operate as a molecular signpost for productive immune homing rather than merely as a generic inflammatory mediator. The functional consequence of CCL5 signaling, however, depends heavily on which CCR5 + cell populations dominate the chemotactic landscape [50]. When CCR5 is preferentially expressed on cytotoxic lymphocytes, CCL5 tends to promote antitumor immunity by enhancing recruitment of CD8^+^T cells and NK cells, both of which can execute direct tumor-cell killing through perforin/granzyme pathways. In this effector context, CCL5 helps to convert immunologic activation into spatial entry, thereby allowing antigen-specific T cells to accumulate within the tumor parenchyma rather than remaining excluded at the periphery [50]. The relevance of this process extends beyond simple cell number. Productive CCL5-associated recruitment also depends on endothelial activation and the availability of adhesion molecules such as ICAM-1 and VCAM-1, which support rolling-to-arrest transitions, firm adhesion, and transmigration across tumor-associated vasculature [51]. Thus, the biological effect of CCL5 is contingent not only on receptor expression by immune cells but also on the competence of the vascular interface to interpret the chemokine cue. Yet the same axis can be co-opted by suppressive compartments of the tumor microenvironment. CCL5 is also implicated in the recruitment of tumor-associated macrophages and, importantly, myeloid-derived suppressor cells [51]. This branch of signaling is potentially consequential because CCR5⁺ myeloid populations can, in selected tumor contexts, blunt CD8⁺ T-cell function, attenuate antigen presentation, promote tissue remodeling, and sustain a wound-like inflammatory niche that supports tumor persistence [19, 24, 41, 47, 51, 52]. In such settings, high CCL5 should not be interpreted as evidence of productive immunity; it may instead accompany myeloid infiltration, immune dampening, and tumor persistence [19, 24, 41, 47, 51, 52]. This explains why the same chemokine can correlate with either favorable immune infiltration or unfavorable tumor biology depending on which cellular compartment dominates receptor availability. The pathway is therefore best understood as a context-sensitive routing system, not a unidirectional antitumor mechanism. This ambiguity is particularly important when considering exercise, because the exercise response may alter the cellular balance that determines whether CCL5–CCR5 signaling is functionally beneficial or detrimental [19, 24, 41, 47, 51, 52].

The present evidence does not support a simple model in which exercise directly and reproducibly increases or decreases CCL5 concentration or CCR5 expression in tumors [53]. Rather, exercise is more plausibly interpreted as a modifier of the biological context in which the pathway operates. Acute exercise induces a transient mobilization of circulating leukocytes, including cells with immediate effector potential, whereas repeated exercise sessions produce longer-term adaptation in immune-cell composition and function [53]. Exercise also increases catecholamine signaling, modulates IL-6 release, and can shift macrophage polarization toward a less suppressive phenotype [54]. These systemic changes do not prove CCL5-specific regulation, but they create a milieu in which CCR5-expressing effector cells may be more abundant, more activated, and more capable of responding to a pre-existing or exercise-permissive CCL5 gradient within the tumor [54]. A second indirect mechanism involves the vascular and stromal compartment. Tumor infiltration by CCR5 + cells requires more than chemokine production; it requires vascular accessibility, endothelial adhesion competence, and a permissive interstitial architecture [54]. Exercise has been reported to improve vascularization, blood flow, and oxygen delivery in tumors, while reducing hypoxia and thereby attenuating HIF-1 -linked barriers to immune entry. Although these effects have not been shown to act specifically through CCL5–CCR5, they are highly relevant to the pathway’s functional efficacy [19, 24, 41, 47, 51–54]. A CCL5 gradient can only recruit cells if the cells can reach the tumor endothelium and if the endothelial barrier is capable of responding by expressing adhesion molecules and supporting transendothelial migration. By improving perfusion and reducing hypoxia-associated vascular dysfunction, exercise may increase the probability that CCR5 + effector cells successfully decode the chemokine signal [19, 24, 41, 47, 51–54]. In this sense, exercise may not rewrite the CCL5 program itself; it may make the program biologically executable. A third layer of indirect remodeling concerns the broader inflammatory network in which CCL5 is embedded [19, 24, 41, 47, 51–54]. Exercise can alter IL-6, IFN- , and catecholamine signaling, all of which influence immune-cell migration and activation states. IFN-γ, in particular, promotes a type 1 inflammatory environment that favors cytotoxic recruitment and can shift the tumor toward a more permissive chemokine profile [39, 54]. Accordingly, the most defensible interpretation is that exercise may exert a context-dependent permissive effect on the CCL5–CCR5 axis. In tumors where CCR5 is enriched on effector lymphocytes and the vascular and stromal compartments are permissive, exercise could facilitate cytotoxic trafficking by increasing immune-cell availability or improving endothelial access. In tumors where CCR5 is predominantly expressed on suppressive myeloid cells, the same chemokine architecture may remain unfavorable, and exercise may have limited capacity to reverse this polarity unless it also alters the myeloid compartment [28–33]. For example, if exercise enhances CXCR3-ligand programs while simultaneously reducing suppressive myeloid recruitment, then CCL5 may become part of a more favorable trafficking network rather than a standalone determinant of tumor behavior. Importantly, the data available here do not justify claiming that exercise consistently remodels CCL5 in humans or in tumor tissue [28–33].

Accordingly, the most defensible interpretation is that exercise exerts a context-dependent permissive effect on the CCL5–CCR5 axis. In tumors where CCR5 is enriched on effector lymphocytes and the vascular/stromal compartment is receptive, exercise may facilitate cytotoxic trafficking by increasing immune-cell availability and improving endothelial access. In tumors where CCR5 is predominantly expressed on suppressive myeloid cells, the same chemokine architecture may remain unfavorable, and exercise may have limited ability to reverse that polarity unless it also reshapes the myeloid compartment [28–33]. This duality is precisely why CCL5–CCR5 deserves separate treatment in a review of exercise and chemokines: it is not simply an “activation” pathway, but a molecular decision node whose outcome depends on which cellular readers are present in the tumor microenvironment. Overall, exercise should be regarded as a potential modulator of CCL5–CCR5 functionality rather than a proven direct regulator of CCL5 abundance. Its likely impact is indirect but biologically meaningful: by mobilizing effector cells, improving perfusion, reducing hypoxia, shifting macrophage polarization, and altering inflammatory tone, exercise may increase the probability that CCR5-mediated trafficking becomes productive in the antitumor direction [19, 24, 41, 47, 51–54]. However, because the same axis can also recruit MDSCs and tumor-associated macrophages, the therapeutic interpretation must remain conditional. The pathway is best framed as a context-sensitive chemokine switch whose output depends on the cellular source of CCL5, the dominant CCR5-expressing populations, and the vascular and stromal state of the tumor. That is the scientifically honest conclusion—and also the more interesting one [19, 24, 41, 47, 51–54]. Supplementary Table S8 synthesizes the dual trafficking logic of the CCL5–CCR5 axis and the exercise-related factors that may shift its net biological output toward either productive antitumor homing or persistent immune suppression.

### Counteracting retention and exclusion signals: exercise effects on the CXCL12–CXCR4 axis

If the CXCL9/CXCL10/CXCL11–CXCR3 system can be viewed as a chemokine program that promotes productive effector-cell entry into inflamed tumors, the CXCL12–CXCR4 axis often represents a more structurally complex and, in many tumors, more restrictive signaling network [55]. Unlike chemokine pathways that primarily guide cytotoxic recruitment into permissive tissue, CXCL12–CXCR4 frequently operates at the interface of stromal retention, endothelial patterning, metastatic tropism, and compartmentalized immune exclusion [55]. Its biological importance lies not only in its potency as a chemoattractant but also in its capacity to create spatial asymmetries within tissues—directing where cells accumulate, where they are retained, and whether they are allowed to access the tumor core or remain trapped within stromal or perivascular niches. In the context of tumor immunology, this makes CXCL12–CXCR4 less a simple recruitment axis than a regulatory system that can determine whether leukocyte trafficking remains superficial and nonproductive or becomes parenchymal and functionally relevant [56]. Under physiological conditions, CXCL12 is a major homeostatic chemokine involved in hematopoietic cell retention, tissue organization, and stem-cell trafficking, with CXCR4 serving as one of its principal receptors on immune and progenitor populations [56, 57]. Within tumors, however, this homeostatic system is frequently repurposed by malignant and stromal compartments. Cancer-associated fibroblasts, endothelial cells, and in some settings tumor cells themselves can generate CXCL12-rich niches that shape both tumor behavior and immune positioning. Rather than simply attracting beneficial immune cells, these gradients may retain leukocytes in stromal regions, promote compartmentalized accumulation outside the tumor nest, or guide suppressive and reparative populations into the microenvironment [56, 57]. The consequence is that a chemokine classically associated with directed migration may become, in the tumor setting, a mediator of spatial misdirection [56, 57]. Cells are recruited, but not necessarily to the place where cytotoxic function can be effectively executed. This exclusionary role is especially relevant in desmoplastic and fibroblast-rich tumors, where CXCL12 contributes to the construction of a stromal barrier that limits penetration of CD8^+^T cells into malignant islets [56, 57]. Mechanistically, this does not require complete absence of immune cells. On the contrary, CXCL12-rich tumors may contain substantial leukocyte populations that remain sequestered within the peripheral stroma or perivascular regions, unable to achieve direct tumor-cell contact. In this regard, the CXCL12–CXCR4 axis exemplifies a critical principle in chemokine biology: trafficking failure is not always caused by insufficient recruitment; it may also arise from mislocalization, retention, or chemotactic diversion [56, 57]. For a review concerned with the molecular syntax of chemokine gradients, this is central, because the problem is not merely whether a gradient exists, but what spatial instruction that gradient encodes.

From an exercise-biology perspective, the key question is whether physical activity can weaken retention-dominant chemokine structures or reduce the stromal and vascular conditions that sustain CXCL12-driven exclusion. Although the available evidence is largely systemic rather than intratumoral, it remains mechanistically relevant because it shows that exercise training can alter one of the chemokines most closely linked to immune retention and exclusion [58–60]. Repeated exercise may therefore shift the balance of cues that favor immune-cell positioning, even if direct evidence for local tumor remodeling is still lacking. A reduction in circulating or systemic CXCL12 does not, by itself, prove that fibroblast-derived intratumoral CXCL12 niches are disrupted or that T cells are released from stromal trapping within tumors [58–60]. That distinction needs to remain clear. That said, the evidentiary boundary must be drawn carefully [60]. A reduction in circulating or systemic CXCL12 does not by itself demonstrate disruption of fibroblast-derived intratumoral CXCL12 niches, nor does it prove relief of stromal T-cell trapping within solid tumors [58–60]. The biology of CXCL12 is highly compartmentalized, and circulating concentrations cannot be assumed to mirror matrix-bound, locally presented, or fibroblast-anchored chemokine signals within the tumor microenvironment [58–60]. Therefore, a rigorous interpretation is that exercise has been shown to influence the broader CXCL12 milieu, but direct proof of exercise-mediated dismantling of intratumoral CXCL12 exclusion zones remains limited. This is precisely where many reviews become sloppy: they convert systemic signal into local certainty. That move is not defensible unless direct tissue-level data exist [58–60]. Even so, there are several biologically credible mechanisms through which exercise could counteract CXCL12–CXCR4-mediated retention. First, exercise improves tumor vascularization, blood flow, and oxygen delivery while reducing hypoxia [58–60]. These changes are not trivial accessories to chemokine biology; they alter the structural context in which chemokines are produced, immobilized, and interpreted [58–60]. Hypoxia sustains fibroblast activation, abnormal extracellular matrix turnover, and endothelial dysfunction, all of which can reinforce exclusionary stromal organization. By reducing hypoxia and improving perfusion, exercise may attenuate some of the environmental conditions that favor persistent CXCL12-rich stromal barriers [58–60]. In other words, exercise may not need to directly suppress CXCL12 transcription to weaken the functional consequence of the axis; it may do so by destabilizing the tissue state that allows CXCL12-dependent exclusion to dominate [56–60]. Second, exercise may influence the cellular sources and readers of CXCL12 signaling [56–60]. Tumor-associated fibroblasts are major producers of extracellular matrix components and stromal chemokines, while myeloid cells and lymphocytes express varying levels of CXCR4 depending on activation state and inflammatory context. If chronic exercise shifts macrophage polarization, reduces suppressive myeloid burden, or enhances the prevalence of activated effector lymphocytes, then the trafficking network through which CXCL12 operates may be altered even without dramatic changes in ligand abundance [56–60]. This point is mechanistically subtle but important: the effect of a chemokine is determined not only by how much ligand is present, but also by who is listening. A tumor rich in CXCL12 but increasingly populated by activated CXCR3^+^cytotoxic cells, improved vascular adhesion cues, and reduced suppressive stromal pressure may interpret the same chemokine field differently than a hypoxic, myeloid-dominant tumor [56–60]. Third, exercise may alter the balance between retention-promoting and infiltration-promoting chemokine axes. A tumor microenvironment is never governed by CXCL12 alone. The biological outcome emerges from competition, hierarchy, and spatial overlap among multiple chemokine systems. If exercise strengthens interferon-linked recruitment pathways such as CXCL9/CXCL10/CXCL11–CXCR3 while simultaneously reducing systemic CXCL12 or attenuating hypoxia-associated stromal dysfunction, then the relative weight of exclusionary signaling may decline even if CXCL12 is not abolished [56–60]. This framing is more realistic than an all-or-none model. Exercise is unlikely to “switch off” CXCL12 in most tumors; what it may do is shift the chemokine balance so that retention cues become less dominant relative to cues that support effector-cell entry and tissue penetration. The acute-versus-chronic distinction is particularly important here [56–60]. Acute exercise is highly effective at transiently mobilizing immune cells, but mobilization alone is insufficient to overcome a CXCL12-structured exclusion barrier [61]. Cells can be made available in the circulation within minutes, yet the stromal logic of the tumor remains largely unchanged over that timescale. By contrast, repeated training may gradually reshape inflammatory tone, perfusion, endothelial competence, and perhaps systemic chemokine set points, thereby affecting retention-dominant pathways such as CXCL12–CXCR4 [61]. If exercise can weaken the functional strength of this pathway—even indirectly—it may enhance the value of other immunological interventions by reducing spatial sequestration of effector cells [61]. This would be particularly important in tumors characterized by stromal exclusion rather than complete immune absence, where the therapeutic problem is not generating T cells but enabling them to cross the final anatomical and chemotactic barriers to tumor-cell contact [61, 62]. In such settings, exercise might serve less as a direct chemokine inducer and more as a physiological strategy for softening exclusionary tissue logic [61, 62]. Nevertheless, scientific honesty requires restraint. The current evidence does not show that exercise directly suppresses fibroblast-derived intratumoral CXCL12, reverses CXCR4-mediated immune trapping in situ, or uniformly reprograms all CXCL12-dependent compartments. The most defensible conclusion is narrower but still important: exercise training can modulate CXCL12 at the systemic level, and through vascular normalization, reduced hypoxia, and inflammatory reorganization, it plausibly weakens the conditions that permit CXCL12–CXCR4-driven retention and exclusion to dominate [61, 62]. This interpretation preserves mechanistic coherence without overstating what has been directly demonstrated. Accordingly, the CXCL12–CXCR4 axis should be positioned in this review as a retention and exclusion program that exercise may partially counterbalance rather than fully reverse [61, 62]. Its significance lies in highlighting that antitumor immunity depends not only on attracting immune cells, but also on preventing their sequestration in nonproductive tissue compartments. If the CXCR3 axis defines one route by which exercise may enhance entry, the CXCL12 axis defines another route by which exercise may relieve entrapment. Together, these pathways underscore the broader thesis that exercise-mediated immune benefit in cancer is fundamentally spatial: it changes not just how many immune cells exist, but where they can go, where they stop, and whether they ultimately reach malignant tissue in a functionally meaningful way [56–62]. Systemic CXCL12 measurements cannot be interpreted as direct evidence of disrupted fibroblast-derived, matrix-bound intratumoral CXCL12 gradients or relief of stromal T-cell sequestration. The spatial determinants of CXCL12–CXCR4-mediated retention and exclusion, together with the vascular, stromal, and exercise-responsive factors that may weaken its functional dominance, are summarized in Supplementary Table S9.

### Acute versus chronic exercise in chemokine regulation: signaling, transcriptional, and epigenetic adaptations

The immune response to exercise is not uniform across time. Acute exercise and repeated training appear to engage partly distinct biological programs: acute bouts primarily induce transient hemodynamic and leukocyte-mobilization responses, whereas repeated exercise may contribute to longer-term adaptations in immune regulation and systemic chemokine tone. This distinction is important when considering whether exercise acts mainly as a short-lived stimulus for immune-cell trafficking or may gradually modify the host conditions under which chemokine signals operate. Serum measurements provide useful but indirect information about these processes. Although acute exercise is accompanied by rapid changes in catecholaminergic, glucocorticoid, and myokine signaling, including IL-6 responses, direct evidence that a single exercise bout remodels chemokine concentrations within tumors remains limited. Accordingly, the following discussion distinguishes established systemic responses from hypotheses concerning chronic exercise-mediated remodeling of the tumor microenvironment [63]. This distinction matters when asking whether exercise acts only as a short-lived trigger for immune trafficking or whether it can gradually shape the structure of chemokine signaling. Current evidence suggests that serum measurements provide useful but indirect insight into these processes. Acute exercise is characterized by rapid changes in catecholamines, glucocorticoids, and muscle-derived myokines such as IL-6, which together drive transient leukocytosis. In that setting, the dominant effect is mobilization [63].

Acute exercise induces a transient systemic response involving catecholaminergic and glucocorticoid signaling, myokine release, altered shear stress, and redistribution of circulating leukocytes [64, 65]. This response mobilizes effector populations, particularly NK cells and activated CD8⁺ T cells, from lymphoid and peripheral compartments into the circulation [64, 65]. In the context of the TME, such mobilization may increase the availability of cells capable of responding to pre-existing intratumoral chemokine gradients. However, human studies indicate that a single exercise session does not consistently produce measurable remodeling of circulating chemokine concentrations [64–66]. Therefore, any immediate effect of acute exercise on antitumor immune surveillance should be interpreted primarily as a consequence of transient changes in leukocyte availability and vascular transit, rather than as evidence of rapid transcriptional reprogramming of the chemokine environment or enhanced tumor infiltration [64–66].

By contrast, repeated exercise may produce more durable adaptations in vascular function, inflammatory tone, and systemic chemokine regulation, although the magnitude, direction, and tissue relevance of these changes depend on exercise dose, training duration, participant characteristics, and disease context [67]. In a short-term intervention such as the two-week training period reported by Clifford et al. [62], repeated exercise was associated with changes in circulating chemokines, including CCL2 and CXCL12. These findings suggest that repeated exposure may influence systemic chemokine tone, but they do not establish altered chemokine production or remodeling within the tumor microenvironment [62]. This distinction is mechanistically significant: whereas acute exercise temporarily boosts the supply of immune cells, chronic exercise may alter the context in which these cells interact with the TME [62]. The systemic reduction of chemokines such as CXCL12 and CCL2 following chronic training—particularly observed after high-intensity protocols—is particularly provocative when viewed through the lens of tumor stroma biology [68]. While these data reflect systemic changes, they imply that the inflammatory milieu is being recalibrated, potentially reducing the dominance of signals that promote stromal retention or suppressive myeloid recruitment [68]. In tumors where a CXCL12-dependent stromal barrier contributes to T-cell exclusion, a training-associated reduction in circulating CXCL12 could, in principle, coexist with changes in vascular or stromal permissiveness that facilitate effector-cell entry. However, this proposed relationship has not been demonstrated by paired measurements of circulating and intratumoral chemokines after exercise [68]. This would represent a fundamental shift in the “molecular syntax” of the TME: the tumor is no longer surrounded by an environment optimized for immune exclusion, but rather by one that permits a more balanced trafficking profile [61–69]. It must be explicitly stated, however, that the jump from systemic chemokine modulation to intratumoral remodeling remains a significant evidentiary boundary [61–69]. Serum chemokine concentrations provide a snapshot of the host’s systemic state, not necessarily the local concentrations within the tumor parenchyma, which are often dictated by tissue-specific fibroblasts, endothelial cells, and malignant cell metabolism [61–69]. The reduction of CXCL12 in circulation does not automatically equate to the dismantling of a fibroblast-anchored chemokine niche within the tumor nest. Nevertheless, the clinical relevance of chronic exercise training is likely rooted in this exact intersection: by repeatedly shifting the systemic chemokine architecture and improving the host’s vascular and inflammatory homeostasis, training may gradually weaken the stromal barriers that prevent effector cells from translating increased availability into deep intratumoral penetration [42, 46, 58, 60–69]. Accordingly, the most defensible distinction is that acute exercise primarily functions as a transient leukocyte-mobilization stimulus, whereas repeated training may influence the systemic, vascular, and inflammatory conditions that regulate immune-cell access to the TME. Whether this constitutes genuine intratumoral remodeling remains to be determined [42, 46, 58, 60–69]. Future studies must determine whether the intensity-dependent systemic chemokine shifts observed after chronic training correlate with quantitative changes in intratumoral chemokine expression. Until such direct evidence is obtained, exercise should be positioned as an intervention that likely potentiates the function of endogenous chemokine gradients through the normalization of the host’s systemic and vascular landscape, rather than as a direct, acute-acting driver of intratumoral chemokine transcription. This distinction between the “burst” of mobilization and the “shift” of adaptation is foundational for integrating exercise into the molecular management of the tumor microenvironment [42, 46, 58, 60–69].

This temporal distinction between acute mobilization and chronic remodeling also raises a deeper mechanistic question: why should repeated exercise produce effects that persist beyond the short-lived hemodynamic and neuroendocrine perturbations of a single bout? The most plausible answer is that chronic training does not merely repeat acute immune activation [70]; rather, it progressively reprograms the transcriptional and, potentially, epigenetic architecture through which inflammatory and chemotactic signals are generated, sensed, and stabilized [71]. In this framework, the durability of exercise-induced immunological benefit is unlikely to be explained by recurrent leukocyte mobilization alone. Instead, repeated exercise may gradually shift the regulatory set points that determine chemokine production, receptor availability, endothelial responsiveness, stromal behavior, and the inflammatory tone of the host–tumor interface [72]. At the transcriptional level, chronic exercise repeatedly exposes immune, endothelial, stromal, and metabolic compartments to oscillatory bursts of catecholamines, glucocorticoids, myokines, altered shear stress, and fluctuating oxygen demand. Although each individual bout is transient, their cumulative effect may entrain more stable patterns of gene regulation [72]. This is particularly relevant for chemokine biology because chemokine networks are highly sensitive to upstream transcriptional programs governed by inflammatory regulators such as NF- κ B, STATs, HIF-1, interferon-responsive factors, and stress-sensitive signaling nodes [73]. Acute exercise may transiently perturb these regulators, but chronic exercise is more likely to alter their baseline activation thresholds, tissue responsiveness, and context-dependent dominance [42, 46, 58, 60–69]. Such a shift would help explain why repeated training can generate chemokine changes detectable at rest, whereas a single exercise session often cannot. This logic is especially compelling in the case of chemokines that are embedded in broader inflammatory circuits rather than released as isolated mediators. CCL2, CXCL12, and related trafficking signals are not simply switched on or off by one stimulus; they are regulated within a matrix of stromal activation, immune-cell cross-talk, hypoxic signaling, endothelial state, and metabolic stress [42, 46, 58, 60–69] Chronic exercise, by repeatedly modifying these upstream determinants, may progressively move the system toward a different transcriptional equilibrium [72]. Under such conditions, reduced basal expression of retention- or recruitment-associated chemokines in circulation may reflect not a fleeting suppression, but a recalibrated inflammatory phenotype [72]. This provides a mechanistic explanation for why repeated training, rather than acute exercise, is more likely to influence chemokine tone in a sustained manner. An epigenetic dimension may further reinforce this adaptive stability [70–73]. Repeated exercise has long been recognized as a physiological stimulus capable of inducing durable molecular memory across multiple tissues, including skeletal muscle, vascular endothelium, and immune compartments [70–73]. Although the current exercise-oncology literature does not yet provide direct, tumor-resolved proof that exercise rewrites methylation marks at chemokine loci within the TME, it is biologically plausible that chronic training influences DNA methylation, histone accessibility, chromatin organization, and enhancer activity in ways that indirectly shape cytokine and chemokine expression programs [70–73]. In this view, exercise training may gradually alter not only which inflammatory genes are expressed, but how readily they can be re-induced, suppressed, or coordinated with other trafficking pathways in response to stress, tumor signals, or immunotherapy [70–73]. Such a possibility is particularly relevant for understanding the persistence of altered inflammatory set points after repeated training. If chronic exercise modifies the epigenetic permissiveness of transcriptional programs linked to hypoxia, endothelial dysfunction, stromal activation, or suppressive myeloid recruitment, then its effects on chemokine signaling may outlast the final exercise bout itself [70–73]. This is conceptually important because it distinguishes training adaptation from serial acute exposure. Acute exercise can transiently increase immune-cell availability, but it does not necessarily create molecular memory [62, 67, 68]. Chronic exercise, by contrast, may induce a form of physiological imprinting in which the host becomes less prone to sustaining exclusionary inflammatory states and more capable of supporting coordinated immune trafficking [70–73]. Even if direct methylation data for tumor chemokines remain sparse, this framework provides a biologically coherent explanation for why training-related effects are more durable, more systemic, and potentially more therapeutically meaningful than the transient leukocytosis induced by a single session [62, 67, 68, 70–73]. The vascular compartment offers a useful example of this principle. Endothelial adhesion competence, inflammatory sensing, and responsiveness to chemotactic cues are not purely immediate phenomena; they are shaped by repeated exposure to flow dynamics, oxidative balance, inflammatory mediators, and tissue oxygenation [62, 67, 68, 70–73]. Chronic exercise may therefore recondition the endothelium at both transcriptional and epigenetic levels, rendering it more permissive to immune-cell tethering, adhesion, and transmigration when appropriate chemokine signals are present [71–73]. Similarly, stromal cells repeatedly exposed to an exercise-modified systemic milieu may become less supportive of persistent hypoxia-driven, exclusionary programs. In this sense, the chronic exercise effect on chemokine biology may be indirect yet structurally deep: it may not require direct transcriptional activation of a single chemokine gene if it instead redefines the tissue logic that determines how chemokine gradients are built, immobilized, and interpreted [71–73]. The same argument applies to immune-cell intrinsic programming. Repeated exercise may influence not only ligand abundance but also receptor expression, migratory competence, and the transcriptional readiness of effector populations to respond to chemotactic cues [71–73]. A chronically trained host may therefore differ from an acutely exercised host not simply by having more circulating immune cells at a given time point, but by possessing immune cells that are phenotypically better prepared to decode inflammatory and chemotactic information. This distinction matters because effective antitumor trafficking requires a coordinated system: ligands must be present, barriers must be permissive, and leukocytes must be competent readers of the spatial code [71–73]. Chronic exercise is uniquely positioned to influence all three layers over time. Nevertheless, caution remains essential. At present, the epigenetic argument is mechanistically persuasive but empirically incomplete in the specific context of tumor chemokine remodeling. The available evidence more strongly supports the existence of chronic exercise-induced systemic adaptation than direct demonstration of methylation changes at chemokine promoters or enhancers within tumors. Therefore, this concept should be framed as a plausible explanatory layer rather than a settled conclusion. The value of including it in the present review is not to claim definitive proof, but to explain why the chronic exercise phenotype is unlikely to be reducible to repeated acute bouts alone [71–73]. Persistent changes in chemokine signaling would be expected to involve sustained alterations in regulation, cell composition, tissue structure, or combinations of these mechanisms. Taken together, these observations suggest that the distinction between acute and chronic exercise is not merely quantitative but qualitatively mechanistic. Acute exercise acts primarily through transient mobilization and short-lived inflammatory perturbation, whereas chronic exercise may gradually impose a more durable transcriptional and potentially epigenetic reorganization of the host–tumor interface [71–73]. This longer-timescale reprogramming offers the most coherent explanation for why repeated training can produce measurable changes in circulating chemokines and more sustained improvements in immune trafficking, vascular permissiveness, and inflammatory balance. Within the broader molecular syntax of the TME, chronic exercise may therefore be understood not simply as a repeated stimulus, but as a biological editor of the rules by which chemokine signals are generated, stabilized, and ultimately translated into immune-cell position [71–73]. The epigenetic component of this model is therefore plausible but empirically incomplete. Current evidence more strongly supports chronic exercise-associated systemic adaptation than direct demonstration of epigenetic remodeling at chemokine promoters, enhancers, or regulatory regions within tumors. The hypothesis is nevertheless experimentally testable through paired analyses of circulating and intratumoral chemokines, cell-type-resolved chromatin accessibility, DNA methylation, histone modifications, and transcriptional activity before and after defined training protocols. Taken together, the distinction between acute and chronic exercise is mechanistic rather than merely temporal. Acute exercise primarily induces transient leukocyte mobilization and short-lived neuroendocrine and hemodynamic changes. Repeated training may produce more durable adaptations in systemic inflammatory regulation, vascular function, immune-cell composition, and, potentially, transcriptional or epigenetic state. However, these systemic adaptations should not be equated with demonstrated intratumoral chemokine remodeling. The most defensible interpretation is that acute exercise may transiently increase the availability of chemokine-responsive immune cells, whereas chronic training may alter the systemic and vascular conditions that influence their access to the TME. The distinction between acute mobilization, chronic systemic adaptation, and putative tumor-resolved remodeling is summarized in Supplementary Table S10.

## Integrating exerkines with chemokine dynamics: a unified model

**Fig. 2:**
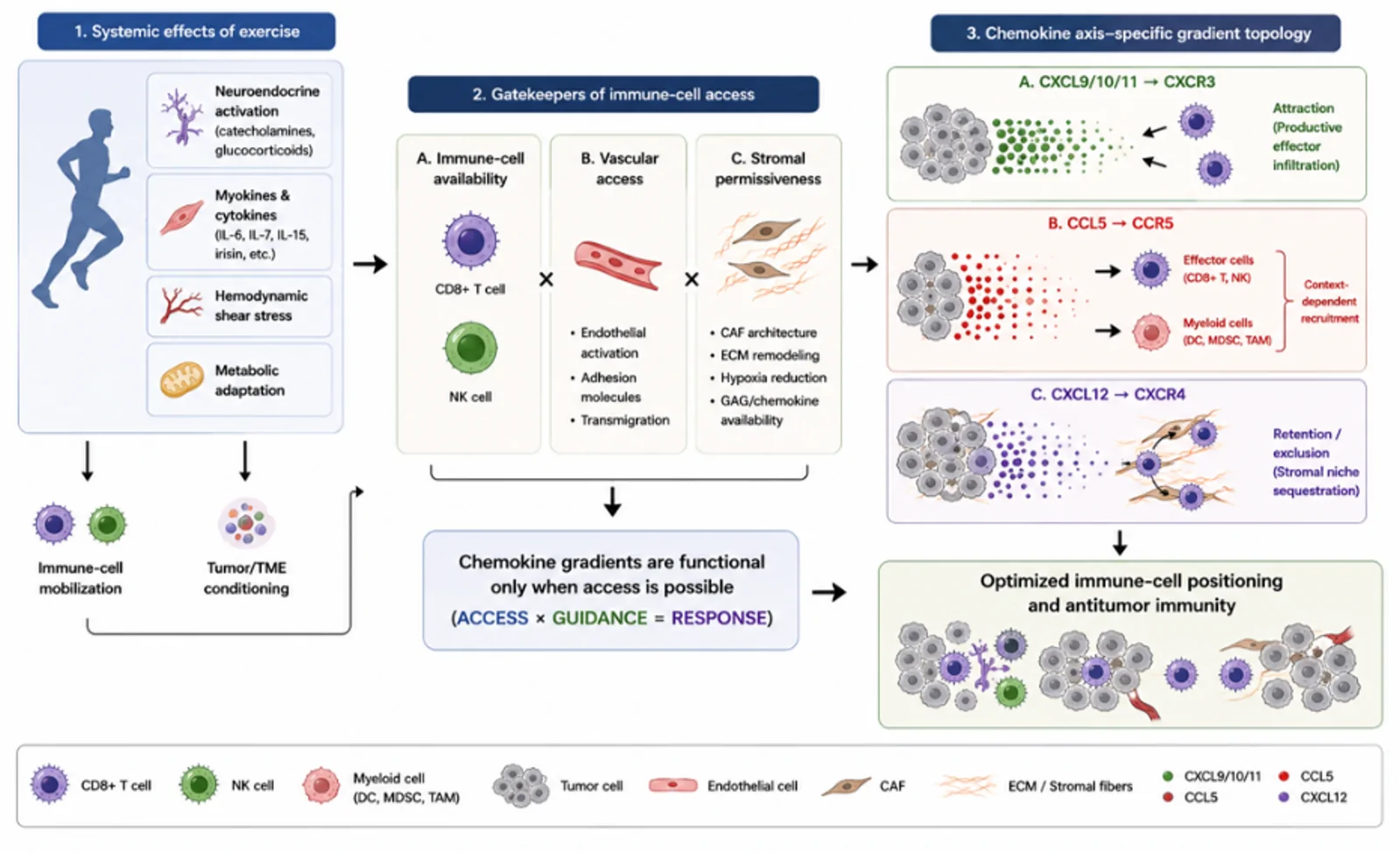
Exercise–chemokine gradient coupling model linking systemic adaptation to intratumoral immune positioning. Exercise generates systemic neuroendocrine, myokine/cytokine, hemodynamic, and metabolic signals that influence both immune-cell mobilization and tumor microenvironment (TME) conditioning. Effective immune-cell homing is proposed to depend on three interconnected gatekeepers: immune-cell availability, vascular access, and stromal permissiveness. Together, these determine whether immune cells can access and interpret chemokine gradients within tumors. The CXCL9/10/11–CXCR3 axis primarily supports productive effector-cell recruitment, the CCL5–CCR5 axis promotes context-dependent recruitment of effector and myeloid populations, and the CXCL12–CXCR4 axis may contribute to stromal retention and immune exclusion. Thus, exercise may enhance antitumor immunity not simply by increasing chemokine abundance, but by coupling immune-cell availability with vascular and stromal conditions that enable chemokine-guided positioning. The model is conceptual and highlights proposed mechanisms requiring direct spatial and mechanistic validation

A coherent understanding of exercise-oncology requires moving beyond the idea that exercise acts on the tumor microenvironment through a single mediator or isolated pathway [74]. Instead, exercise should be conceptualized as a systemic physiological perturbation that releases a coordinated set of exerkines, neuroendocrine signals, and metabolic adaptations, each of which can influence how chemokine gradients are generated, perceived, and translated into immune-cell trafficking [1–7, 34, 74]. In this unified model, exerkines do not replace chemokines; rather, they reshape the conditions under which chemokines become functionally meaningful [1–7, 34, 74, 75]. The principal consequence of exercise is therefore not the direct synthesis of a specific chemokine axis, but the reorganization of the host–tumor interface so that existing chemokine cues are more effectively interpreted by effector leukocytes [1–7, 34, 74, 75]. Among the most relevant exercise-induced mediators are skeletal muscle-derived myokines, particularly IL-6 and IL-15, together with other exercise-associated factors such as irisin, decorin, IL-10, and IL-8. These molecules are context dependent, and their effects cannot be reduced to a simple pro- or anti-tumor classification. IL-15 is especially notable because of its role in supporting the proliferation, maturation, and functional competence of T cells and NK cells [1–7, 34, 62, 68–75]. From the perspective of chemokine biology, this is important because chemokine gradients are only effective when a sufficiently large and competent pool of circulating effector cells exists to respond to them. Thus, one of the central ways exercise may amplify chemokine-driven immunity is by increasing the number and quality of cells capable of responding to CXCL9/10/11–CXCR3, CCL5–CCR5, and related axes [1–7, 34, 62, 68–75]. A second layer of this model involves acute neuroendocrine mobilization. Exercise induces transient increases in catecholamines and glucocorticoids, which can rapidly redistribute circulating leukocytes and mobilize cells with immediate effector potential, particularly NK cells and cytotoxic T cells. This acute response should not be confused with a direct change in intratumoral chemokine production [1–7, 34, 62, 68–75]. Rather, it increases the availability of responsive immune cells in the circulation, thereby improving the likelihood that these cells will encounter and follow chemokine cues in the tumor bed. In this way, catecholamine-driven mobilization acts upstream of chemokine-guided trafficking, augmenting the cellular substrate on which chemokine gradients operate. The vascular compartment constitutes a third and essential layer. Chemokine gradients do not act in a vacuum; they must be interpreted across an endothelial barrier that can either facilitate or obstruct leukocyte entryv [1–7, 34, 62, 68–75]. Exercise promotes vascular perfusion, endothelial nitric oxide bioavailability, and, in some settings, vascular normalization. These effects improve oxygen delivery, reduce hypoxia, and may diminish lactate accumulation within the tumor microenvironment [1–7, 34, 62, 68–75]. Because hypoxia and metabolic stress are strongly linked to immunosuppressive transcriptional programs, their reduction can indirectly improve the accessibility and functional clarity of chemokine signals. In practical terms, even a chemokine gradient that already exists may become more biologically effective if the endothelial barrier becomes more permissive and the stromal environment less exclusionary [1–7, 34, 62, 68–75]. This point is especially relevant for the CXCL12–CXCR4 axis, which is deeply involved in stromal retention, immune exclusion, and cell entrapment. If exercise lowers hypoxia, improves perfusion, and attenuates the stromal conditions that maintain chemokine sequestration, then the biological consequence may be a reduction in immune-cell trapping and a greater capacity for effector cells to traverse the tumor stroma [1–7, 34, 62, 68–75]. Similarly, for CXCL9/10/11–CXCR3, improved vascular access and greater effector-cell availability may enhance the recruitment of IFN- responsive T cells and NK cells, even if the chemokine concentration itself changes only modestly [1–7, 34, 62, 68–75]. For CCL5–CCR5, the picture is more context dependent: the same axis may support antitumor effector trafficking in one setting while recruiting suppressive myeloid cells in another. Exercise may therefore alter not only the magnitude of chemokine signaling but also its cellular meaning by changing the composition of the responding leukocyte pool. This compositional effect is central to any unified model of exerkines and chemokines [1–7, 34, 62, 68–75]. Exercise has been associated with increased intratumoral CD8^+^T-cell infiltration, greater memory CD8^+^ representation, reduced Tregs, and a shift of tumor-associated macrophages toward a more M1-like phenotype [1–7, 34, 62, 68–75]. These changes suggest that exercise modifies the immune ecosystem in a way that biases chemokine signaling toward productive antitumor recruitment rather than suppressive or futile trafficking. In other words, the same chemokine gradient may have different consequences depending on whether the tissue contains mostly exhausted, suppressive, or cytotoxic responder populations. Exercise appears to push the system toward the latter. The available human data reinforce the plausibility of this model [1–7, 34, 62, 68–77]. A particularly useful way to synthesize these observations is to view exerkines as signal amplifiers and recontextualizers rather than as direct chemokine substitutes. Myokines such as IL-6 and IL-15 can expand or activate effector-cell compartments; catecholamines can mobilize them into circulation; vascular adaptation can improve tissue access; and reduced hypoxia/lactate stress can weaken the suppressive architecture of the TME [1–7, 34, 62, 68–75]. Chemokines then function as the spatial code that channels these newly available effector cells into the tumor. The biological output is therefore emergent: it depends on the alignment of systemic readiness, endothelial permissiveness, stromal architecture, and local chemokine instruction. This perspective also clarifies why exercise effects are often modest when examined through a single molecular lens but more convincing when studied as a systems phenomenon [1–7, 34, 62, 68–75]. A lone exerkine rarely explains the entire phenotype because exercise is not a single-molecule therapy. It is a coordinated physiological state that changes ligand availability, receptor competence, vascular structure, and inflammatory set points simultaneously. Chemokine dynamics in the TME should therefore be interpreted as one downstream expression of this broader state transition. The most defensible conclusion is not that exercise directly induces a specific chemokine program, but that it establishes a permissive immunological and stromal environment in which chemokine programs become more effective, less suppressive, and more aligned with antitumor trafficking. Accordingly, the unified model proposed here can be summarized as follows: exercise triggers a multilayered exerkine response involving myokines, catecholamines, and vascular mediators; these signals enhance effector-cell abundance and competence, improve endothelial access, and reduce hypoxia-associated suppression; and these systemic and stromal changes together increase the functional impact of tumor chemokine gradients such as CCL5–CCR5, CXCL9/10/11–CXCR3, and CXCL12–CXCR4. In this framework, exercise acts not as a direct chemokine inducer, but as a physiological editor of the chemokine landscape, shaping how trafficking signals are produced, presented, and ultimately translated into immune-cell infiltration [1–7, 34, 62, 68–75]. Table 2 and Graphic abstract and 2 (Fig. 2).

**Table 2:** Exercise-mediated remodeling of the tumor chemokine landscape: an integrated mechanistic framework

Level / Axis	Core biological role in TME	How exercise may act	Direct vs. indirect evidence	Functional implication for immune trafficking	Strength of evidence / caveat
Systemic exercise response	Sets the upstream physiological context for chemokine signaling	Acute catecholamine/glucocorticoid surges, myokine release, transient leukocytosis, altered perfusion	Direct for mobilization; indirect for tumor chemokine remodeling	Expands the circulating pool of effector cells capable of responding to tumor chemokines	Strong for mobilization; limited for intratumoral chemokine transcription
Exerkines: IL-6	Inflammatory and metabolic myokine with context-dependent immune effects	Exercise-induced IL-6 may support immune activation, cytotoxic competence, and inflammatory recalibration	Direct for exercise-induced release; indirect for chemokine modulation	May increase readiness of effector cells and favor a more inflamed trafficking environment	Context dependent; not a tumor chemokine-specific regulator
Exerkines: IL-15	Promotes T-cell and NK-cell proliferation, maturation, and cytotoxic potential	Exercise-associated IL-15 may expand the pool of CXCR3- and CCR5-responsive effector cells	Direct for muscle-derived immunoregulatory role; indirect for chemokine-linked infiltration	Increases the number and quality of cells available for chemokine-guided tumor entry	Strong mechanistic plausibility; limited direct TME mapping
Catecholamines	Acute systemic mobilization signal	Exercise transiently redistributes NK cells and cytotoxic T cells into circulation	Direct for mobilization; not direct for chemokine production	Enhances the availability of immune cells that can decode tumor chemokine gradients	Primarily acute, not sufficient alone for durable remodeling
Vascular normalization / endothelial competence	Governs whether chemokines can be functionally translated into entry	Exercise improves blood flow, endothelial NO bioavailability, adhesion competence, and perfusion	Indirect but strongly mechanistically supported	Makes chemokine cues more “readable” at the endothelial interface and improves transmigration	Very important mediator; not a ligand-level effect
Hypoxia / lactate stress	Drives immunosuppressive, exclusionary, and stromal programs	Exercise may reduce hypoxia and lactate burden through improved perfusion and oxygen delivery	Indirect	Weakens exclusionary stromal logic and supports productive chemokine interpretation	Plausible, but tumor-specific metabolic proof remains limited
CXCL9/10/11–CXCR3 axis	Core interferon-linked effector-cell recruitment program	Exercise may strengthen type 1 inflammatory context, improve endothelial access, and support CXCR3 + lymphocyte infiltration	Indirect with some preclinical support	Promotes CD8^+^ T cell and NK-cell trafficking into inflamed tumors	Strongest axis for exercise-linked antitumor trafficking, but direct intratumoral upregulation remains limited
CCL5–CCR5 axis	Ambivalent routing system for effector and suppressive cells	Exercise may improve the functional context in which CCR5 + cells respond to CCL5	Indirect, context dependent	Can enhance cytotoxic trafficking or, in some tumors, recruit suppressive myeloid cells	Must be interpreted by dominant CCR5 + compartment; not uniformly beneficial
CXCL12–CXCR4 axis	Retention, stromal sequestration, and immune exclusion program	Repeated exercise may reduce systemic CXCL12, improve perfusion, and weaken exclusionary stromal logic	Direct for circulating CXCL12 change; indirect for intratumoral exclusion	May reduce immune trapping and facilitate deeper T-cell penetration	Human evidence is systemic; intratumoral proof remains sparse
CCL2 and monocyte/macrophage trafficking	Associated with myeloid recruitment and suppressive remodeling	Exercise-associated reductions in CCL2 may attenuate recruitment of inflammatory monocytes	Direct in serum; indirect in TME	Potentially reduces suppressive myeloid influx and supports a more favorable immune composition	Important secondary axis, but not the central trafficking route for effector lymphocytes
Macrophage polarization	Shapes chemokine production, scavenging, and stromal interpretation	Exercise may shift TAMs toward a more M1-like phenotype and reduce suppressive tone	Indirect, supported by preclinical data	Reorients the chemokine milieu toward antitumor recruitment rather than wound-healing programs	Likely critical for axis-level context dependence
CD8 + T-cell infiltration	Final functional readout of productive chemokine guidance	Exercise increases intratumoral CD8^+^ T-cell abundance and memory fraction in some models	Direct in preclinical reports; mechanism inferred	Indicates that systemic mobilization is translated into actual tumor entry	Important outcome, but not proof of one specific chemokine axis
NK-cell trafficking and competence	Rapid effector compartment that responds to chemokine cues	Exercise increases NK-cell mobilization and may enhance receptor expression and infiltration	Direct for mobilization and some receptor changes; indirect for chemokine coupling	Improves immediate cytotoxic surveillance and chemokine responsiveness	Strong in animal/preclinical literature
Transendothelial migration / adhesion molecules	Endothelial checkpoint for chemokine-guided entry	Exercise may increase vascular permissiveness and facilitate ICAM-1/VCAM-1-dependent entry	Indirect	Converts circulating immune availability into tissue penetration	Critical mechanistic bridge often ignored in over-simplified models
Acute exercise	Short-lived mobilization state	Catecholamine-driven leukocytosis and transient immune redistribution	Direct for mobilization	Increases encounter probability between effector cells and existing chemokine gradients	Generally insufficient to remodel chemokine set points
Chronic exercise	Durable physiological adaptation	Repeated training alters inflammatory tone, vascular state, myeloid balance, and chemokine set points	Indirect but more durable	Supports sustained infiltration-permissive TME remodeling	More likely than acute exercise to produce meaningful chemokine-level effects
Transcriptional reprogramming	Determines chemokine availability and receptor readiness	Chronic exercise may alter NF-κB, STAT, HIF-1α, and interferon-linked signaling thresholds	Mechanistically plausible; not definitively tumor-proven	Could shift baseline chemokine production and responsiveness	Strong explanatory framework, but avoid overclaiming
Epigenetic reprogramming	May stabilize longer-term changes in inflammatory memory	Exercise may modify chromatin accessibility, methylation, enhancer activity, and gene inducibility	Hypothesis-generating	Provides a possible basis for persistent remodeling after training	Must be framed as a plausible layer, not settled fact
Unified model	Integrates systemic, vascular, stromal, and chemokine levels	Exercise acts as a multiscale physiological editor of the host–tumor interface	Synthesized inference	Improves the probability that chemokine gradients become spatially effective	Most defensible interpretation given current evidence

Exercise acts as a multiscale physiological perturbation that reshapes chemokine-guided immune trafficking through coordinated effects on exerkines, catecholamine signaling, vascular permissiveness, stromal organization, immune-cell composition, and metabolic stress. Rather than directly inducing a single chemokine axis, exercise recalibrates the conditions under which chemokine cues become spatially and functionally interpretable within the tumor microenvironment. Direct evidence is strongest for acute immune mobilization and selected systemic chemokine changes, whereas intratumoral remodeling of chemokine gradients remains largely indirect and context dependent.

## Discussion

Although the precise cellular and molecular mechanisms by which exercise remodels tumor immunity remain incompletely defined, further dissection of these pathways is likely to sharpen the mechanistic basis for exercise recommendations in cancer care. Within this emerging framework, exercise should not be viewed simply as a systemic perturbation that transiently mobilizes leukocytes or alters a narrow panel of inflammatory mediators. Rather, it is more appropriately understood as a multiscale physiological regulator capable of influencing the TME at transcriptional, vascular, stromal, metabolic, and spatial levels. This distinction is fundamental because the immunological consequence of exercise cannot be inferred from leukocyte mobilization or chemokine abundance alone. Chemokine networks regulate the trafficking, retention, positioning, and activation of diverse immune populations, including cytotoxic T cells, NK cells, dendritic cells, macrophages, neutrophils, and other lymphoid and myeloid subsets. These populations may exert antitumor, tissue-remodeling, immunoregulatory, or protumor functions depending on their differentiation state, activation profile, spatial localization, and interactions with tumor, stromal, vascular, and lymphatic compartments. Accordingly, effective immune recruitment should be defined not simply by an increase in the number of recruited cells, but by the establishment of a spatially organized and functionally productive immune response. This requires the tumor to present appropriate chemokine signals, maintain them within an infiltration-permissive tissue architecture, and provide endothelial and stromal conditions that support productive entry, positioning, and activity within the parenchyma.

In this framework, the tumor microenvironment is not a passive recipient of immune-cell movement. It acts as a selective barrier and organizational niche that can retain, exclude, redirect, or permit different immune populations depending on the chemokine signals and structural constraints present within it. Exercise may influence this system by shifting the relative strength and accessibility of these signals; however, the direction and biological significance of such changes cannot be predicted from total leukocyte influx alone. The most plausible interpretation is not that physical activity induces a single antitumor chemokine, but that it may alter the balance and functional meaning of several chemokine pathways simultaneously. The same chemokine axis may promote the entry of cytotoxic lymphocytes in one tumor context while supporting the accumulation, retention, or functional activity of suppressive myeloid cells in another. This is especially relevant in immune-excluded and immune-desert tumors, where effector cells may be mobilized systemically but fail to penetrate the tumor core because of endothelial anergy, stromal sequestration, abnormal perfusion, hypoxia, dense extracellular matrix, and suppressive myeloid programs. Conversely, cells that successfully enter the tumor may remain functionally ineffective or may contribute to immunosuppression if they acquire an exhausted, suppressive, dysfunctional, or tissue-remodeling phenotype. A useful way to understand exercise-induced remodeling is therefore to distinguish acute mobilization from chronic remodeling and to assess both processes in a lineage- and compartment-resolved manner. Acute exercise can rapidly increase the availability of circulating NK cells, CD8 + T cells, and other leukocyte populations through catecholaminergic and hemodynamic mechanisms. Nevertheless, mobilization alone is not equivalent to tumor infiltration, appropriate spatial localization, or effective antitumor activity. Importantly, this blood-vessel-centered model of immune access should be expanded to include the lymphatic compartment. Tumor-associated lymphatic vessels are not passive drainage structures; they actively regulate interstitial fluid clearance, tissue pressure, chemokine distribution, antigen transport, dendritic-cell migration, lymphocyte trafficking, and communication between the tumor and tumor-draining lymph nodes. These functions may influence different immune populations in distinct ways. For example, lymphatic transport can support dendritic-cell migration and antigen delivery to draining lymph nodes, whereas altered lymphatic–stromal interactions may also contribute to the redistribution or retention of suppressive myeloid populations within the tumor. Lymphatic remodeling and lymphangiogenesis can therefore influence not only the magnitude of immune-cell recruitment but also the composition, spatial organization, and functional quality of antitumor immune priming. This consideration is particularly relevant to the CCL19/CCL21–CCR7 axis, through which lymphatic and lymphoid niches help coordinate the migration and positioning of dendritic cells and CCR7-expressing lymphocytes. However, the consequences of these processes are context dependent and should not be interpreted as uniformly beneficial without determining which cell populations are recruited, where they localize, and whether they acquire functionally antitumor or immunosuppressive states. This is where chronic training may become particularly relevant: repeated exercise may gradually influence vascular adhesion, perfusion, oxygenation, stromal composition, and myeloid-cell states. However, such remodeling should not be presumed to improve immune function uniformly. Changes in myeloid polarization, for example, may either reduce suppressive programs or alter the abundance and activity of macrophage and neutrophil subsets with context-dependent effects. Therefore, the potential benefit of chronic exercise depends not only on whether more immune cells enter the tumor, but also on whether the resulting cellular composition, spatial distribution, and functional state favor effective antitumor immunity. Among the chemokine axes most relevant to this concept, the CXCL9/10/11–CXCR3 pathway is likely the most directly linked to productive anti-tumor T-cell trafficking. These chemokines are classically associated with type 1 inflammatory programs and IFN-linked signaling. Exercise may support this axis indirectly by reinforcing an inflammatory milieu that favors CXCR3 + effector cell recruitment, especially when combined with therapies that already amplify interferon-driven immune activation. Yet the available evidence remains insufficient to claim that exercise robustly and uniformly increases intratumoral CXCL9, CXCL10, or CXCL11 in humans. The safer and more defensible interpretation is that exercise may create a physiological context in which this axis becomes more effective and more spatially consequential. The CCL5–CCR5 axis requires even more caution. It is tempting to frame CCL5 as a beneficial homing signal, but that would be an oversimplification. CCL5 can support T-cell and NK-cell trafficking, yet it can also participate in recruitment of suppressive myeloid populations or other protumor circuits depending on context. Therefore, any increase in CCL5 should not automatically be interpreted as favorable. The biological meaning of this axis depends on the full immune contexture, vascular accessibility, and the balance between effector and suppressive lineages. Exercise may modulate this pathway primarily by changing the environment in which it operates rather than by directly and reproducibly altering ligand abundance in all tumors. The CXCL12–CXCR4 axis is likely an important counterweight to productive immune infiltration, but its effects should also be interpreted in a cell- and compartment-specific manner. CXCL12-rich niches can retain immune cells within stromal or perivascular compartments and limit their penetration into malignant islets. This axis is closely linked to fibroblast activity, extracellular-matrix organization, hypoxia, vascular remodeling, and the recruitment or retention of suppressive myeloid populations. However, CXCL12–CXCR4 signaling may not produce a uniform outcome across all responding cells; its consequences depend on which immune or stromal populations express CXCR4 and how the resulting cells are positioned within the TME. In this sense, exercise may not merely increase recruitment signals; it may alter the balance between permissive trafficking and exclusionary retention. Yet a reduction in retention or an increase in tissue entry should not be assumed to be beneficial unless it is accompanied by a favorable shift in immune-cell composition and function. These exclusionary processes may also intersect with lymphatic remodeling, because altered drainage and lymphatic–stromal interactions can influence the spatial retention, redistribution, and presentation of chemokines within the TME. If so, the therapeutic value of exercise may partly involve shifting the spatial balance from suppressive sequestration toward functionally productive infiltration, rather than simply increasing the total number of immune cells within the tumor. The strongest conceptual contribution of this manuscript is therefore the proposal that exercise may alter signal production, signal display, signal accessibility, and signal interpretation simultaneously. Importantly, the relevant outcome is not a generalized increase in chemokine signaling or immune-cell abundance. Rather, the key issue is whether exercise changes the composition, spatial organization, and functional state of the immune infiltrate in a direction that supports tumor control. This is a more defensible framework than claiming that exercise simply upregulates one chemokine or downregulates another. In practical terms, exercise may help transform a tumor from a chemically and physically exclusionary site into a more infiltration-permissive landscape, but such a transformation should be considered beneficial only when recruited cells are appropriately localized and functionally competent. The same remodeling may be neutral or even adverse if it preferentially supports suppressive myeloid populations, regulatory lymphocytes, dysfunctional neutrophils, or other protumor programs. This effect is therefore likely to be lineage-specific, axis-specific, dose-dependent, time-dependent, spatially heterogeneous, and highly contingent on the underlying tumor phenotype.

### **Clinical and translational recommendations**

If exercise is to be positioned as a serious component of immuno-oncology, the field must move beyond generic recommendations and adopt a precision translation framework. At minimum, the data suggest that exercise should not be treated as a universal adjuvant with a uniform mechanism across all tumor types. Instead, its implementation should be guided by baseline tumor immune contexture, vascular phenotype, stromal density, and the degree of immune exclusion.

First, exercise prescription should be context specific. Tumors with an inflamed phenotype are mechanistically different from immune-excluded or immune-desert tumors. In the former, exercise may mainly amplify pre-existing immune activity; in the latter, it’s more important role may be to improve vascular permissiveness, reduce stromal barriers, and restore productive chemokine gradients. This means that exercise protocols should not be evaluated as one-size-fits-all interventions. Intensity, frequency, duration, and timing relative to therapy likely matter, and these variables must be reported rigorously. Second, clinical trials should expand beyond peripheral biomarkers. Measuring only circulating cytokines, leukocyte counts, or plasma chemokines is not sufficient to establish mechanistic relevance. Those measurements may reflect systemic mobilization, but they do not prove intratumoral infiltration or functional immune restoration. Future trials should incorporate spatial analyses of tumor tissue, ideally combining multiplex imaging, transcriptomics, and proteomic mapping to determine whether exercise changes chemokine distribution at the invasive margin, within the stroma, and in the tumor core. These analyses should also include the lymphatic compartment. Assessment of lymphatic density, lymphatic endothelial activation, lymphangiogenic remodeling, interstitial fluid handling, and tumor-draining lymph node communication could help determine whether exercise modifies immune access through mechanisms that are not captured by blood-vessel measurements alone. Where feasible, spatial profiling should map chemokines such as CCL19 and CCL21 relative to lymphatic vessels, dendritic cells, CCR7-expressing lymphocytes, blood vessels, and stromal regions. The key translational question is therefore not whether a ligand changes in blood, but whether exercise reorganizes the vascular–lymphatic–stromal network in a manner that improves immune access, antigen transport, lymphoid communication, and functional immune-cell positioning within the tissue. Third, exercise should be considered a rational primer for combination therapy. The most plausible clinical utility is not as monotherapy, but as a physiological enhancer of existing treatments. In combination with immune checkpoint blockade, exercise may improve the recruitment and localization of CXCR3 effector cells. In combination with vascular normalization strategies, it may strengthen endothelial adhesion and improve trafficking efficiency. In combination with stromal-targeting interventions, it may help dismantle collagen-rich barriers and release immune cells from retention niches. This combinatorial logic is strong, but it should still be tested empirically rather than assumed.

Fourth, exercise dose must be standardized with greater precision. Too many studies still describe exercise in vague terms that make mechanistic interpretation impossible. To identify a true dose-response relationship, interventions should report intensity relative to VO2max or heart rate reserve, session duration, frequency, cumulative training load, and sampling time relative to the last bout. Acute and chronic responses must not be conflated. Without temporal precision, mechanistic conclusions about chemokines will remain weak.

Finally, clinicians and translational researchers should avoid a simplistic “more chemokine is better” interpretation. The biological effect of exercise depends on whether a given axis promotes effector recruitment or suppressive retention. In other words, the clinically meaningful endpoint is not altered ligand concentration per se, but improved immune access, reduced exclusion, and enhanced anti-tumor function within the tissue.

## **Future directions**

Despite the conceptual appeal of this framework, several major gaps remain. Addressing them is essential before exercise can be fully integrated into precision immuno-oncology.

The first priority is spatial and lineage-resolved validation. Future studies should measure chemokines directly inside tumors rather than relying only on circulating markers. Multiplex imaging, spatial transcriptomics, and spatial proteomics could be used to determine whether exercise changes the distribution of CXCL9/10/11, CCL5, CXCL12, CCL19, and CCL21 relative to blood vessels, lymphatic vessels, stromal regions, tumor nests, and immune infiltrates. These approaches should resolve not only the density of infiltrating cells but also the identity and functional state of cytotoxic T cells, NK cells, regulatory lymphocytes, dendritic cells, macrophage and monocyte subsets, neutrophil populations, and other relevant immune lineages. The same spatial framework should explicitly resolve lymphatic vessels and lymphatic-associated immune niches. Future studies should determine whether exercise alters lymphatic density, morphology, endothelial activation, drainage capacity, or lymphangiogenesis, and whether these changes are associated with the localization of CCL19, CCL21, CCR7-expressing lymphocytes, dendritic cells, antitumor effector populations, and suppressive myeloid cells. Parallel assessment of tumor-draining lymph nodes will also be important, because changes in intratumoral lymphatic transport may influence antigen delivery and the initiation or maintenance of adaptive antitumor immunity. The key question is not simply whether a chemokine is detectable or whether more immune cells are present, but whether its spatial pattern produces a functionally productive and compositionally favorable immune infiltrate. Many current associations are plausible, but they remain unproven. Controlled preclinical systems, including organoids, tumor-on-chip platforms, and co-culture models with endothelial and stromal components, will be needed to test whether specific exerkines directly regulate chemokine programs. The third priority is time resolution. Acute and chronic exercise are likely to produce different chemokine signatures, and longitudinal sampling will be necessary to distinguish short-term mobilization from longer-term remodeling. The fourth priority is cell-type resolution. The same chemokine axis may act differently depending on which cells express the ligand or receptor. For example, CCL5–CCR5 could support either anti-tumor lymphocyte recruitment or suppressive myeloid trafficking. Similarly, CXCL12 can function as a retention cue in stromal niches or as part of broader vascular and metastatic programs. Lineage-resolved studies are therefore necessary to determine which cells are actually responding to exercise-induced changes. The fifth priority is integration of metabolic biology. Exercise changes lactate handling, hypoxia, perfusion, redox state, and tissue fluid dynamics, all of which may influence chemokine signaling and lymphatic function. It is entirely plausible that exercise-induced reductions in hypoxia and lactate, together with changes in interstitial fluid handling, reshape HIF-dependent transcription, lymphatic endothelial behavior, and chemokine retention, thereby contributing to the conversion of the TME from an exclusionary to a recruitment-permissive state. This metabolic-chemokine crosstalk deserves much more attention, because it may explain why some tumors respond to exercise while others do not. The sixth priority is epigenetic and transcriptional memory. If chronic exercise induces stable changes in chromatin accessibility, enhancer activity, or DNA methylation within TME-resident cells, then exercise may produce durable reprogramming rather than only transient modulation. This could help explain prolonged changes in immune accessibility even after the acute effects of a session have faded. Investigating these memory-like effects should be a major future direction. Lastly, future research must determine which patients are most likely to benefit. Tumor type, baseline immune exclusion, stromal architecture, obesity status, cachexia, treatment history, and concurrent therapy may all influence responsiveness. A precision framework is needed to identify where exercise is likely to be immunologically meaningful and where it may have limited impact.

A particularly important next step is the simultaneous, spatially resolved assessment of immune cells, chemokines, and both blood and lymphatic vessels across distinct intratumoral compartments, especially the invasive margin, stroma, and tumor core. This is essential because these regions differ markedly in vascular access, stromal density, lymphatic organization, and immune exclusion, and therefore may respond differently to exercise-induced remodeling. Future studies should not only quantify chemokine abundance, but also determine whether their spatial distribution aligns with the localization of specific immune lineages and vascular structures that govern cellular trafficking and tissue entry. Multiplex imaging, spatial transcriptomics, and spatial proteomics will be particularly valuable for defining whether exercise reshapes chemokine gradients in a way that promotes productive immune infiltration, rather than simply increasing detectable immune-cell abundance. In parallel, mapping blood-vessel and lymphatic-vessel architecture will help clarify whether exercise alters vascular accessibility, lymphatic drainage, or immune-cell positioning within these compartments. Together, these spatially integrated analyses will be necessary to establish whether exercise meaningfully converts the tumor microenvironment into a more permissive niche for anti-tumor immunity.

## Concluding perspective

Overall, the present framework supports a more rigorous and biologically realistic view of exercise in cancer: not as a generic anti-inflammatory intervention or a uniform enhancer of immune-cell recruitment, but as a context-dependent regulator of immune accessibility and immune organization. Its principal value may lie in remodeling the chemokine landscape, improving vascular and lymphatic permissiveness, reducing physical and molecular exclusion, and influencing the composition, localization, and functional state of immune cells within the TME. However, increased immune-cell recruitment should be considered beneficial only when it results in appropriately localized and functionally competent antitumor responses. The same chemokine remodeling may have different or opposing consequences depending on whether it favors cytotoxic lymphocytes, competent antigen-presenting cells, suppressive macrophages, regulatory lymphocytes, neutrophil subsets, or other immune populations. The real translational promise of exercise oncology is therefore not simply that it may increase immune access, but that it may help organize the vascular–lymphatic–stromal and chemokine networks in a manner that supports effective tumor control. Whether this occurs will depend on tumor phenotype, exercise dose and timing, treatment context, and the identity and functional state of the recruited cells.

## Supplementary Information

Below is the link to the electronic supplementary material.

Supplementary Material 1

## Data Availability

No datasets were generated or analysed during the current study.
