# Supplementary material for: Exercise-induced systemic and tumor-microenvironment adaptations regulating chemokine-guided immune-cell trafficking
Source: Clin Exp Med. 2026 Sep 1;26(1):381. doi: 10.1007/s10238-026-02315-z (PMC13768373; doi:10.1007/s10238-026-02315-z)
Supplement: Supplementary file 1 [file 10238_2026_2315_MOESM1_ESM.docx]

**Appendix**

**Supplementary Methods and Tables**

**Exercise-induced systemic and tumor-microenvironment adaptations regulating chemokine-guided immune-cell trafficking**

Contents

[Section 1. Supplementary Methods	3](#_Toc237251710)

[Table S1. Core Search Domains and Representative Search Terms	12](#_Toc237251711)

[Table S2. Representative Search Combinations	13](#_Toc237251712)

[Figure S1. Graphic abstract.	14](#_Toc237251713)

[Table S3. Molecular Syntax, Structural Features, and Exercise-Induced Regulatory Potentials of Key TME Chemokine Axes	15](#_Toc237251714)

[Table S4. Biophysical Mechanisms, Signaling Dynamics, and Spatial Organization of Chemokine Gradients in the Tumor Microenvironment (TME)	16](#_Toc237251715)

[Table S5. Multilayered Mechanisms Underlying Failure of Immune-Cell Homing in Solid Tumors	18](#_Toc237251716)

[Table S6. Putative Mechanisms by Which Exercise Remodels the Tumor Chemokine Landscape	21](#_Toc237251717)

[Table S7. The CXCL9/CXCL10/CXCL11–CXCR3 Axis: Molecular Checkpoints and Exercise-Mediated Restoration of Antitumor Homing	24](#_Toc237251718)

[Table S8. Context-Dependent Remodeling of the CCL5–CCR5 Axis by Exercise	25](#_Toc237251719)

[Table S9. The CXCL12–CXCR4 Axis as a Retention and Immune-Exclusion Program: Exercise-Relevant Mechanisms and Evidentiary Boundaries	28](#_Toc237251720)

[Table S10. Acute versus Chronic Exercise as Distinct Regulatory Programs in Chemokine Biology: Signaling, Transcriptional, Epigenetic, and Trafficking-Relevant Adaptations	31](#_Toc237251721)

# Section 1. Supplementary Methods

**1.1. Scope of the Review**

This article was developed as a narrative and mechanistic review examining how exercise may influence immune-cell trafficking within the tumor microenvironment (TME). The review focused on the potential interactions among exercise-induced systemic responses, circulating immune-cell mobilization, endothelial and stromal permissiveness, tumor-associated chemokine networks, and the metabolic and oxygenation conditions that regulate immune-cell migration and function.

Particular emphasis was placed on three chemokine axes with established or potentially relevant implications for immune-cell trafficking in cancer:

1. the CXCL9/CXCL10/CXCL11–CXCR3 axis, which is primarily associated with the recruitment and positioning of activated and cytotoxic T-cell populations;
2. the CCL5–CCR5 axis, whose effects may be context-dependent and may involve both effector lymphocyte recruitment and the trafficking of selected myeloid or immunosuppressive populations; and
3. the CXCL12–CXCR4 axis, which is associated with stromal retention, cellular sequestration, hypoxia-related signaling, and immune exclusion.

The review also considered the possibility that exercise may influence chemokine-guided trafficking indirectly by modifying vascular perfusion, endothelial activation, stromal architecture, tissue oxygenation, lactate accumulation, redox status, and local immunometabolic conditions. The central interpretive premise was that exercise-induced changes in circulating immune cells or soluble chemokines cannot automatically be equated with improved intratumoral immune infiltration. Accordingly, systemic, vascular, stromal, metabolic, and intratumoral evidence were evaluated as related but distinct levels of biological organization.

**1.2. Literature Search and Narrative Review Approach**

A structured literature search was undertaken to identify studies relevant to the effects of exercise on immune-cell trafficking, chemokine signaling, tumor–immune interactions, and the metabolic regulation of the TME. Because the present article was designed as a narrative and integrative review rather than a systematic review or meta-analysis, the search strategy was intended to capture a broad range of physiological, immunological, mechanistic, translational, and exercise-oncology evidence. The literature search was conducted from database inception through 9 August 2026. The primary databases consulted were PubMed/MEDLINE, Scopus, and Web of Science. Additional relevant sources were identified through ScienceDirect, Google Scholar, and manual screening of the reference lists of eligible original studies, mechanistic investigations, and relevant review articles.

The search strategy combined terms related to exercise, high-intensity interval training, cancer, the tumor microenvironment, chemokines, chemokine receptors, immune-cell trafficking, vascular and stromal regulation, and immunometabolism. The principal search terms included:

- “exercise oncology”
- “exercise training”
- “acute exercise”
- “chronic exercise”
- “exercise intensity”
- “exercise dose”
- “high-intensity interval training”
- “HIIT”
- “high-intensity intermittent exercise”
- “tumor microenvironment”
- “cancer”
- “tumor immune microenvironment”
- “immune-cell trafficking”
- “immune-cell homing”
- “immune infiltration”
- “T-cell recruitment”
- “natural killer cells”
- “cytotoxic lymphocytes”
- “chemokine”
- “chemokine gradient”
- “chemokine receptor”
- “CXCL9”
- “CXCL10”
- “CXCL11”
- “CXCR3”
- “CCL5”
- “CCR5”
- “CXCL12”
- “CXCR4”
- “endothelial permeability”
- “vascular normalization”
- “stromal exclusion”
- “cancer-associated fibroblasts”
- “hypoxia”
- “lactate”
- “acidosis”
- “immunometabolism”
- “exerkines”
- “cytokines”
- “perfusion”
- “redox state”
- “HIF-1α”
- “hypoxia-inducible signaling”

Boolean operators, including **AND** and **OR**, were applied as appropriate for each database. Representative search combinations included:

- “exercise” AND “tumor microenvironment”
- “exercise” AND “chemokine”
- “exercise” AND “immune-cell trafficking”
- “exercise” AND “CXCL10”
- “exercise” AND “CXCR3”
- “exercise” AND “CCL5”
- “exercise” AND “CXCL12”
- “exercise” AND “CXCR4”
- “high-intensity interval training” AND “immune cells”
- “exercise” AND “tumor infiltration”
- “exercise” AND “lactate” AND “tumor”
- “exercise” AND “hypoxia” AND “chemokine”
- “vascular normalization” AND “exercise” AND “cancer”
- “cancer-associated fibroblasts” AND “CXCL12”
- “chemokine gradient” AND “T-cell migration”

Search terms were adapted to the indexing structure and syntax of each database. No restriction was imposed on publication year or geographical region, allowing the inclusion of both foundational studies and recent developments in exercise immunology, tumor biology, chemokine signaling, and cancer immunometabolism.

**1.3. Eligibility Criteria**

Studies were considered eligible when they examined at least one of the following domains:

1. Exercise-induced systemic immune responses, including changes in circulating leukocyte subsets, natural killer cells, T-cell populations, cytokines, chemokines, or exerkines.
2. Exercise-related modulation of tumor immunity, including changes in immune-cell recruitment, tumor infiltration, immune-cell activation, immune surveillance, or antitumor function.
3. Chemokine and chemokine-receptor signaling relevant to immune-cell migration or positioning within tumors, particularly the CXCL9/10/11–CXCR3, CCL5–CCR5, and CXCL12–CXCR4 axes.
4. Vascular and endothelial mechanisms that may regulate immune-cell access to the tumor, including perfusion, endothelial activation, vascular normalization, adhesion molecules, permeability, and transendothelial migration.
5. Stromal and extracellular-matrix mechanisms, including cancer-associated fibroblasts, stromal barriers, extracellular-matrix organization, chemokine retention, and mechanisms of immune exclusion.
6. Metabolic and oxygenation mechanisms, including hypoxia, lactate accumulation, acidosis, redox regulation, HIF-dependent signaling, adenosine, and other immunometabolic factors that may influence chemokine production or immune-cell responsiveness.
7. Acute or chronic exercise responses, including studies evaluating immediate responses to a single exercise bout and longer-term adaptations following repeated exercise training.

Human studies, animal studies, cellular and molecular investigations, tumor-model studies, exercise-training studies, and relevant physiological, immunological, oncological, and mechanistic reviews were considered when they provided conceptually relevant evidence. Studies involving exercise modalities other than HIIT were retained when they provided relevant information about exercise intensity, immune-cell mobilization, chemokine signaling, vascular or stromal remodeling, tumor immunity, or metabolic mechanisms that could plausibly inform the interpretation of high-intensity exercise. Evidence from non-exercise settings was also considered when it provided mechanistic insight into chemokine regulation, immune-cell migration, hypoxia, lactate signaling, or stromal exclusion.

**1.4. Exclusion Criteria**

Studies were excluded when they met one or more of the following criteria:

1. They were not published in a peer-reviewed source.
2. They were unavailable in sufficient detail for meaningful interpretation.
3. They focused exclusively on exercise performance, body composition, or general health outcomes without a relevant connection to immune regulation, cancer biology, chemokine signaling, or the TME.
4. They examined circulating immune or inflammatory markers without providing a meaningful conceptual link to immune-cell trafficking or tumor-related biology.
5. They evaluated lactate, hypoxia, or acidosis solely as nonspecific clinical or metabolic markers without addressing their potential relevance to immune regulation, chemokine signaling, or tumor biology.
6. They lacked a meaningful conceptual connection with exercise, immune-cell trafficking, chemokine biology, tumor immunity, or the metabolic regulation of the TME.
7. They were editorials, opinion pieces, conference abstracts, or narrative claims unsupported by interpretable primary or secondary evidence.

Studies from pathological contexts, including cancer, sepsis, chronic inflammation, tissue hypoxia, and sustained hyperlactatemia, were included only when they provided mechanistic information relevant to immune-cell migration, chemokine regulation, metabolic stress, or immune suppression. Such evidence was interpreted as contextual or hypothesis-generating rather than as direct evidence of physiological exercise-induced remodeling.

**1.5. Study Identification and Evidence Selection**

Following the search process, titles and abstracts were examined for conceptual relevance to the scope of the review. Full texts were assessed when the relevance of a study could not be determined from the title and abstract or when detailed interpretation of the experimental model and outcome measures was required.

Evidence was selected according to:

- relevance to the central research questions;
- biological and mechanistic plausibility;
- methodological clarity;
- directness of the evidence;
- appropriateness of the exercise model;
- relevance of the cancer or tumor model;
- validity of the immune, chemokine, vascular, stromal, or metabolic outcomes; and
- contribution to the integration of systemic, cellular, tissue-level, and translational perspectives.

The review did not aim to estimate a pooled effect size or to establish a formal quantitative summary of the literature. Instead, the evidence was organized thematically according to the biological level and type of evidence provided.

**1.6. Data Extraction and Evidence Categorization**

Information extracted from relevant studies included:

- first author and publication year;
- study type and experimental model;
- species, cancer type, and tumor model, where applicable;
- participant characteristics and training status;
- exercise modality, intensity, duration, frequency, and training period;
- acute versus chronic exercise design;
- timing of blood, tissue, or tumor sampling;
- immune-cell populations examined;
- chemokines and chemokine receptors assessed;
- circulating versus intratumoral outcomes;
- methods used to quantify immune-cell infiltration or trafficking;
- vascular and endothelial outcomes;
- stromal and extracellular-matrix outcomes;
- metabolic and oxygenation variables;
- lactate, hypoxia, acidosis, or redox-related measures;
- principal molecular or cellular targets;
- major findings;
- methodological limitations; and
- the degree to which the findings supported association, mechanism, or causal inference.

Particular emphasis was placed on whether a study directly measured intratumoral immune-cell trafficking or only assessed circulating immune-cell redistribution or soluble chemokine concentrations. The analysis also considered whether the exercise intervention was sufficiently characterized to permit interpretation of exercise dose and intensity, and whether sampling was performed at a time point capable of distinguishing acute redistribution from longer-term tissue remodeling.

**1.7. Mechanistic Domains Used for Synthesis**

The literature was synthesized across four interconnected mechanistic domains.

**1.7.1. Systemic Immune Mobilization**

This domain included acute exercise-induced changes in circulating leukocyte subsets, natural killer cells, T-cell populations, cytokines, chemokines, and exerkines. These findings were interpreted primarily as evidence of systemic immune mobilization and altered immune availability rather than as direct evidence of enhanced tumor infiltration.

**1.7.2. Vascular and Stromal Permissiveness**

This domain included exercise-related changes in perfusion, endothelial activation, vascular normalization, adhesion molecules, vascular permeability, extracellular-matrix organization, cancer-associated fibroblasts, and stromal barriers. These mechanisms were considered relevant because chemokine gradients may not produce effective immune-cell recruitment unless the vascular and stromal environment permits cellular access.

**1.7.3. Chemokine-Guided Immune-Cell Trafficking**

This domain focused on ligand–receptor axes involved in immune-cell recruitment, retention, or exclusion. Particular attention was given to the CXCL9/10/11–CXCR3, CCL5–CCR5, and CXCL12–CXCR4 pathways. The interpretation considered not only changes in chemokine abundance but also spatial presentation, receptor expression, cellular source, matrix retention, and the functional consequences for immune-cell migration.

**1.7.4. Metabolic and Oxygenation Context**

This domain included hypoxia, lactate accumulation, acidosis, perfusion, redox state, adenosine, and HIF-dependent signaling. These factors were considered potential regulators of both chemokine production and immune-cell responsiveness. Importantly, lactate-rich or hypoxic conditions observed in tumors were not assumed to be equivalent to transient physiological lactate elevations during exercise.

**1.8. Acute Versus Chronic Exercise**

To reduce conceptual ambiguity, studies were classified according to whether they examined:

- **acute exercise**, defined as responses occurring during or shortly after a single exercise bout; or
- **chronic exercise**, defined as adaptations observed after repeated exercise training over time.

Acute exercise findings were interpreted in relation to transient leukocyte redistribution, short-term cytokine and chemokine responses, catecholamine exposure, hemodynamic changes, and temporary changes in vascular or metabolic state. Chronic exercise findings were interpreted in relation to longer-term adaptations in tumor perfusion, vascular function, stromal architecture, immune-cell composition, inflammatory tone, and tumor growth or progression.

Where the timing of outcome assessment was insufficient to distinguish immediate redistribution from sustained tissue remodeling, the evidence was interpreted cautiously.

**1.9. Directness of Evidence and Causal Interpretation**

The strength and directness of evidence were considered according to the following categories:

1. **Direct exercise evidence**: studies in which exercise was experimentally administered and relevant immune, chemokine, vascular, stromal, metabolic, or tumor outcomes were measured.
2. **Mechanistically informative experimental evidence**: cellular, molecular, or animal studies that directly manipulated a pathway relevant to exercise-induced immune-cell trafficking but did not necessarily include an exercise intervention.
3. **Indirect contextual evidence**: studies conducted in pathological or non-exercise settings that provided relevant information about hypoxia, lactate, chemokine signaling, stromal exclusion, or immune-cell migration.
4. **Associative evidence**: studies reporting correlations between exercise exposure, circulating biomarkers, or clinical outcomes without direct manipulation or mechanistic confirmation.
5. **Causal or intervention-based evidence**: studies involving pathway inhibition, receptor blockade, genetic manipulation, or other experimental approaches capable of testing whether a proposed mechanism was necessary or sufficient.

This framework was used to avoid treating changes in circulating chemokine concentrations as proof of altered intratumoral chemokine gradients. Similarly, increased circulating immune-cell numbers were not interpreted as evidence of productive tumor infiltration unless tissue-level or spatially resolved measurements supported that conclusion.

**1.10. Narrative Synthesis**

Given the heterogeneity of the available evidence with respect to cancer type, tumor model, exercise modality, exercise intensity, training duration, sampling time, immune-cell population, chemokine axis, and outcome assessment, a narrative synthesis approach was adopted rather than a quantitative meta-analysis.

The evidence was organized thematically to examine:

1. exercise-induced systemic immune mobilization;
2. the relationship between exercise dose and immune responses;
3. vascular and endothelial mechanisms regulating immune-cell access;
4. stromal and extracellular-matrix barriers to immune infiltration;
5. the CXCL9/10/11–CXCR3 axis;
6. the CCL5–CCR5 axis;
7. the CXCL12–CXCR4 axis;
8. the interaction between chemokine signaling and tumor metabolism;
9. the potential roles of lactate, hypoxia, acidosis, and redox state;
10. the distinction between acute immune redistribution and chronic tissue remodeling; and
11. the translational implications and limitations of exercise-based modulation of tumor immunity.

This approach enabled the integration of direct human evidence with mechanistically informative studies while maintaining a distinction between established findings, plausible biological interpretations, and hypotheses requiring prospective validation.

**1.11. Methodological Quality and Risk of Bias**

Because this article is a narrative review, a formal risk-of-bias instrument or standardized methodological quality score was not systematically applied to every included study. Nevertheless, the interpretation of the literature considered several methodological features, including:

- study design;
- sample size and statistical power;
- participant characteristics and training status;
- cancer type and disease stage;
- tumor-model validity;
- exercise modality and reproducibility;
- exercise intensity, duration, and dose;
- timing and frequency of biological sampling;
- distinction between circulating and intratumoral outcomes;
- validity of immune-cell and chemokine measurements;
- spatial resolution of tissue analyses;
- control of confounding factors;
- use of pathway manipulation or mechanistic controls; and
- directness of the evidence for exercise-induced tumor immune remodeling.

Greater interpretive weight was given to studies that included physiologically relevant exercise protocols, clearly defined sampling schedules, direct tissue-level or spatially resolved measurements, appropriate controls, and experimental manipulation of the proposed pathway.

Particular caution was applied when extrapolating findings from supraphysiological in vitro exposures, isolated-cell systems, non-exercising disease models, or pathological states to the transient physiological responses induced by exercise.

**1.12. Limitations of the Search and Interpretation**

The present review has several methodological limitations. First, because it was designed as a narrative rather than a systematic review, the search and synthesis were not intended to provide an exhaustive or formally reproducible estimate of the total evidence base. Second, substantial heterogeneity existed across studies in terms of cancer type, tumor stage, exercise dose, exercise modality, immune-cell populations, chemokine targets, sampling time, and outcome definitions.

Third, many studies assessed circulating biomarkers or peripheral immune-cell responses without measuring intratumoral chemokine gradients or tissue-level immune-cell localization. Therefore, systemic changes cannot be assumed to reflect local remodeling of the TME. Circulating chemokine concentrations may not mirror matrix-bound, locally presented, or fibroblast-associated chemokine signals within the tumor.

Finally, evidence from pathological settings such as cancer, sepsis, chronic inflammation, sustained hypoxia, or persistent hyperlactatemia was used primarily as mechanistic context. These findings were not interpreted as direct evidence that transient exercise-induced changes in lactate, hypoxia, perfusion, or redox state produce equivalent effects in the tumor. The review therefore distinguishes between observations supported by direct exercise studies and mechanistic inferences that require validation using longitudinal, spatially resolved, and pathway-specific experimental designs.

# Table S1. Core Search Domains and Representative Search Terms

| Search domain | Representative terms |
| --- | --- |
| Exercise and training | exercise, exercise training, acute exercise, chronic exercise, exercise intensity, exercise dose, HIIT, high-intensity interval training, high-intensity intermittent exercise |
| Cancer and TME | cancer, tumor, tumour, tumor microenvironment, tumor immune microenvironment, tumor stroma, cancer-associated fibroblasts |
| Immune-cell trafficking | immune-cell trafficking, immune-cell homing, immune infiltration, T-cell recruitment, natural killer cells, cytotoxic lymphocytes, leukocyte migration |
| Chemokine signaling | chemokine, chemokine gradient, chemokine receptor, CXCL9, CXCL10, CXCL11, CXCR3, CCL5, CCR5, CXCL12, CXCR4 |
| Vascular mechanisms | endothelial activation, endothelial permeability, vascular normalization, tumor perfusion, adhesion molecules, transendothelial migration |
| Stromal mechanisms | stromal exclusion, extracellular matrix, cancer-associated fibroblasts, matrix retention, fibroblast-derived chemokines |
| Metabolic mechanisms | lactate, hypoxia, acidosis, redox state, HIF-1α, immunometabolism, adenosine, metabolic stress |
| Translational context | exercise oncology, cancer immunotherapy, immune surveillance, antitumor immunity, exerkines |

# Table S2. Representative Search Combinations

| Conceptual question | Example search string |
| --- | --- |
| Exercise and chemokines | (“exercise” OR “exercise training”) AND (“chemokine” OR “chemokine gradient”) |
| Exercise and immune trafficking | (“exercise” OR “HIIT”) AND (“immune-cell trafficking” OR “immune infiltration” OR “T-cell recruitment”) |
| Exercise and CXCR3 signaling | (“exercise” OR “exercise training”) AND (“CXCL9” OR “CXCL10” OR “CXCL11” OR “CXCR3”) |
| Exercise and CCL5/CCR5 | (“exercise” OR “exercise training”) AND (“CCL5” OR “CCR5”) |
| Exercise and CXCL12/CXCR4 | (“exercise” OR “exercise training”) AND (“CXCL12” OR “CXCR4”) |
| Exercise and tumor microenvironment | (“exercise” OR “HIIT”) AND (“tumor microenvironment” OR “tumor immunity”) |
| Exercise and metabolic regulation | (“exercise” OR “HIIT”) AND (lactate OR hypoxia OR acidosis OR “redox state”) |
| Metabolism and chemokine signaling | (lactate OR hypoxia OR HIF-1α) AND (chemokine OR “immune-cell migration”) |
| Vascular access and immunity | (“vascular normalization” OR perfusion OR endothelium) AND (“immune infiltration” OR “immune-cell trafficking”) |
| Stromal exclusion | (“cancer-associated fibroblasts” OR stroma OR “extracellular matrix”) AND (CXCL12 OR CXCR4 OR “immune exclusion”) |

# Figure S1. Graphic abstract.

Schematic comparison of acute versus chronic exercise effects on chemokine regulation and immune-cell trafficking in the tumor microenvironment. Acute exercise primarily induces rapid systemic leukocyte mobilization through catecholamine-, glucocorticoid-, and myokine-dependent signaling, increasing the pool of circulating effector cells available to respond to pre-existing tumor trafficking cues. In contrast, chronic exercise training may gradually remodel the vascular, stromal, inflammatory, and transcriptional context in which chemokine pathways operate, thereby shifting the balance between immune-cell exclusion and infiltration. Dashed arrows indicate proposed mechanisms that remain incompletely validated at the intratumoral level. Created with [BioRender.com](http://BioRender.com).

# Table S3. Molecular Syntax, Structural Features, and Exercise-Induced Regulatory Potentials of Key TME Chemokine Axes

| **Chemokine Axis** | **Structural & Biochemical Features** | **Primary TME Role & Spatial Dynamics** | **Exercise-Induced Regulatory Potential** | **Methodological & Mechanistic Limitations** |
| --- | --- | --- | --- | --- |
| **CXCL9, CXCL10, CXCL11 – CXCR3** | • Interferon-inducible CXC ligands.<br>• Share CXCR3 receptor (splice variants CXCR3-A [activating] & CXCR3-B [angiostatic]).<br>• High affinity for glycosaminoglycans (GAGs) creating localized solid-phase gradients. | • Recruitment of $\mathrm{CD}8^{+}$cytotoxic T lymphocytes (CTLs), $Th1$cells, and Natural Killer (NK) cells.<br>• Regulation of intratumoral effector lymphocyte positioning relative to the tumor core. | • Acute exercise: Transient systemic mobilization of $\mathrm{CXCR}3^{+}$cytotoxic effector cells via catecholamine-driven hemodynamics.<br>• Chronic exercise: Potential upregulation of tumor-level CXCL9/10 expressions via IFN-$\gamma$ signaling pathways. | • Circulating ligand concentration does not reflect local matrix-bound gradient density.<br>• Redundancy among CXCL9, 10, and 11 complicates isolating single-ligand exercise effects. |
| **CCL5 – CCR5** | • CC chemokine class.<br>• Exhibits high propensity for self-oligomerization (forming high-molecular-weight polymers).<br>• Binds to GAGs; active as both soluble monomer and immobilized polymer. | • Dual/Context-dependent: Recruits effector $\mathrm{CD}8^{+}$T cells, but also mediates infiltration of immunosuppressive $T_{\mathrm{reg}}$cells, MDSCs, and tumor-promoting $\mathrm{TAMs}$(M2-like). | • Shear stress and systemic myokine signaling may alter endothelial CCL5 presentation.<br>• Exercise-mediated reduction in systemic inflammatory tone may suppress pathological CCL5-driven myeloid recruitment. | • CCR5 expression on opposing cell types (effector vs. suppressor cells) makes the net functional outcome of exercise highly context-dependent. |
| **CXCL12 – CXCR4 / ACKR3** | • Constitutive homeostatic chemokine.<br>• Monomer-dimer equilibrium modulates receptor activation kinetics.<br>• Regulated by atypical chemokine receptor 3 (ACKR3) acting as a decoy receptor/scavenger. | • Retains immune cells within the tumor stroma (immune exclusion).<br>• Promotes cancer-associated fibroblast (CAF) activation, angiogenesis, and tumor cell survival/metastasis. | • Exercise training may alleviate TME hypoxia, potentially downregulating the HIF-1$\alpha$-dependent CXCL12/CXCR4 retention axis.<br>• Potential restoration of ACKR3 scavenging function via improved perfusion. | • Most evidence on CXCL12-mediated exclusion during exercise remains associative; causal pathway blockade studies are scarce in exercise oncology. |
| **General Oligomerization & GAG-Binding Properties** | • Self-association into dimers/tetramers/polymers.<br>• Basic amino acid clusters interact electrostatically with acidic sulfate groups on heparan sulfate proteoglycans. | • Prevents chemokine washing/clearance by interstitial flow.<br>• Creates directional haptotactic tracks (solid-phase gradients) rather than simple chemotactic (liquid-phase) gradients. | • Exercise-induced changes in tumor interstitial fluid pressure (IFP) and shear stress may alter physical chemokine-GAG dissociation kinetics. | • In vitro assays using soluble chemokines fail to capture the biophysical complexity of matrix-bound haptotactic gradients in vivo. |
| **Decoy & Scavenger Receptors (ACKRs)** | • G-protein-independent atypical receptors (e.g., ACKR1 on venules, ACKR3/4 in stroma).<br>• Internalize and degrade chemokines to shape gradient steepness. | • Prevent receptor desensitization of active immune cells.<br>• Control chemokine compartmentalization between blood, stroma, and lymphatics. | • Exercise-induced shear stress and endothelial activation may modulate ACKR1/ACKR3 expression patterns on tumor vasculature. | • ACKRs are technically challenging to quantify and isolate in low-biopsy exercise oncology protocols. |

**Abbreviations**: ACKR: Atypical Chemokine Receptor CAF: Cancer-Associated Fibroblast CCL/CCR: CC Chemokine Ligand/Receptor CTL: Cytotoxic T Lymphocyte CXCL/CXCR: CXC Chemokine Ligand/Receptor GAG: Glycosaminoglycan HIF-1α: Hypoxia-Inducible Factor 1-alpha IFP: Interstitial Fluid Pressure MDSC: Myeloid-Derived Suppressor Cell NK: Natural Killer TAM: Tumor-Associated Macrophage Th1: Type 1 T Helper cell TME: Tumor Microenvironment Note: The exercise-induced regulatory potentials are proposed mechanisms based on the integration of physiological signaling principles (e.g., catecholamine-driven mobilization, shear stress, perfusion changes) and mechanistic findings in non-exercise oncology literature. Dashed arrows or specified limitations indicate areas requiring further experimental validation at the intratumoral level.

# Table S4. Biophysical Mechanisms, Signaling Dynamics, and Spatial Organization of Chemokine Gradients in the Tumor Microenvironment (TME)

| **Mechanism / Phenomenon** | **Biophysical & Molecular Basis** | **TME-Specific Pathology / Context** | **Cellular & Migratory Consequences** | **Potential Exercise-Induced Regulation** |
| --- | --- | --- | --- | --- |
| **GAG Binding & Haptotaxis** | Electrostatic interaction between basic amino acid residues (Lys, Arg) on chemokines and acidic sulfate/carboxyl groups on proteoglycans (e.g., Heparan Sulfate). | High interstitial fluid flow and vascular leakiness in tumors would wash away purely soluble (chemotactic) gradients. | Converts soluble gradients into immobilized, matrix-bound tracks. Essential for directional persistence (haptotactic guidance) and resisting convective loss. | Mechanical shear stress and altered fluid dynamics during exercise may modify GAG synthesis or chemokine-GAG dissociation kinetics. |
| **Monomer-Dimer/Oligomer Equilibrium** | Concentration-dependent self-association of chemokine ligands, stabilized by GAG interaction. | High local production and stromal crowding trap chemokines, driving oligomerization (e.g., CXCL12, CCL5). | Oligomerization facilitates stable presentation on endothelial cells for integrin activation/extravasation. However, excessive dimerization can arrest migration. | Shunting of blood flow and transient pressure changes may disrupt local chemokine depots, altering the oligomerization state. |
| **G-Protein Coupled Receptor (GPCR) Activation** | Two-step model: (1) Chemokine core binds receptor N-terminus (affinity). (2) Chemokine N-terminus inserts into TM bundle, activating $G\alpha_{i}$signaling. | High concentrations of competing ligands and tumor-derived factors saturate receptors. | Dissociation of $G\beta\gamma$activates PI3K and PLC-$\beta$, driving local $\mathrm{PI}P_{3}$accumulation, Rac1/Cdc42 activation at the leading edge, and RhoA-driven rear retraction. | Systemic adrenaline spikes during acute exercise alter GPCR signaling cross-talk via heterologous receptor regulation. |
| **Biased Signaling (e.g., CCR7 Axis)** | Distinct ligands (e.g., CCL19 vs. CCL21) stabilize different active receptor conformations, engaging divergent downstream effectors. | Segregation of CCL19/21 between stroma and lymphatics dictates dendritic cell and T-cell homing. | Differentially regulates $\beta$-arrestin recruitment, receptor internalization, and signal duration, determining whether a cell migrates or arrests. | Chronic training-induced vascular normalization may restore spatial distribution patterns of CCL19 and CCL21. |
| **Homologous & Heterologous Desensitization** | Homologous: GRK-mediated phosphorylation of active receptors recruit $\beta$-arrestin, causing clathrin-mediated endocytosis.<br>Heterologous: PKA/PKC-mediated phosphorylation without ligand. | Abundant inflammatory cytokines and chemokines in the TME induce chronic receptor downregulation. | Prevents receptor saturation, maintains directional sensing. Heterologous desensitization establishes a functional hierarchy among competing cues. | Transient elevations in physiological stressors (e.g., systemic cytokines) dynamically reprogram receptor sensitivity on circulating cells. |
| **Atypical Chemokine Receptors (ACKRs)** | 7-TM receptors (e.g., ACKR3/4) lacking $G\alpha_{i}$coupling. Internalize, degrade, or transcytose ligands. | ACKR3 on tumor or endothelial cells scavenges CXCL12, preventing CXCR4 saturation. | Creates physical ligand sinks that sharpen gradient steepness and prevent receptor desensitization, maintaining migration fidelity. | Perfusion-induced shear stress modulates ACKR expression on blood vessels, regulating local chemokine scavenging rates. |
| **Spatial Macrophage Niche Segregation** | Recruited monocytes ($Ly6C^{+}/CCR2^{+}$) localize to hypoxic cores; resident tissue macrophages ($\mathrm{CD}{206}^{\mathrm{hi}}$) localize to vascular/TLS regions. | Core hypoxia/acidosis vs. perivascular normoxia dictate macrophage phenotype and local chemokine production. | $\mathrm{CD}{206}^{\mathrm{hi}}$macrophages secrete CXCL9/10, organizing effector T/B cell nests in TLS. Recruited TAMs support extracellular matrix remodeling. | Shifting the TME metabolic profile (alleviating hypoxia) via exercise may redirect monocyte recruitment away from immunosuppressive niches. |
| **Immune-Excluded vs. Immune-Desert Phenotypes** | CXCL12-rich stromal barriers trap CXCR4+ lymphocytes at the margin (exclusion). Failure of cDC1 recruitment via CCL4/XCL1 (desert). | Fibroblast-driven matrix densification and chemokine trapping. | Physical trapping of cytotoxic cells in the stroma prevents direct contact with cancer cells, suppressing antitumor immunity. | Shear stress and vascular remodeling modify stromal densification, potentially converting excluded phenotypes into inflamed ones. |

# Table S5. Multilayered Mechanisms Underlying Failure of Immune-Cell Homing in Solid Tumors

| **Barrier / Mechanism** | **Cellular Level** | **Molecular Basis** | **Functional Consequence in the TME** | **Potential Relevance to Exercise** |
| --- | --- | --- | --- | --- |
| **Mislocalized chemokine presentation** | Cancer cells, CAFs, pericytes, endothelial cells | Spatially restricted CXCL12, CCL2, CCL5, CXCL8, and related ligands; strong GAG binding; ligand sequestration at stromal or perivascular sites | Generates retention zones at the invasive margin rather than productive intratumoral gradients; leukocytes accumulate outside tumor nests | Exercise-induced vascular remodeling may alter chemokine exposure at endothelial and stromal interfaces, but direct intratumoral evidence remains limited |
| **Chemokine inactivation / proteolytic editing** | Tumor cells, neutrophils, macrophages, stromal cells | Proteases such as MMPs, cathepsins, and DPP4-like enzymes truncate or modify chemokines; N-terminal cleavage reduces receptor engagement | Converts attractant signals into weak, non-directional, or even antagonistic cues; reduces chemotactic fidelity | Reduced inflammatory burden with training may lower protease-driven chemokine disruption, but causality is not established |
| **Atypical chemokine receptor scavenging** | Endothelial cells, stromal cells, tumor cells | ACKR3, ACKR4, and related receptors internalize or sequester ligands without productive G-protein signaling | Lowers local chemokine availability, sharpens or depletes gradients, and limits leukocyte entry | Improved perfusion/shear stress with exercise may alter ACKR expression or activity, but evidence is indirect |
| **Endothelial anergy / dysfunction** | Tumor endothelial cells | VEGF, endothelin-1, prostaglandins, and chronic inflammatory signaling suppress ICAM-1, VCAM-1, and chemokine presentation | Impairs rolling-to-arrest transition, reduces firm adhesion, and limits transmigration | Exercise may improve endothelial phenotype and perfusion, potentially restoring permissiveness |
| **Abnormal vascular architecture** | Endothelium, pericytes | Irregular branching, poor pericyte coverage, immature vessels, leaky endothelium | Poor delivery of effector cells and unstable endothelial activation; vascular collapse under tumor pressure | Chronic exercise may support vascular normalization and better functional perfusion |
| **Reduced endothelial chemokine display** | Endothelial cells | Defective luminal presentation of chemokines on glycosaminoglycans and surface proteoglycans | Leukocytes cannot receive the activation signal needed for integrin activation | Exercise may increase shear-associated endothelial remodeling, but direct exercise-oncology data are sparse |
| **Stromal fibrosis / ECM densification** | CAFs, fibroblasts, matrix-producing stromal cells | Collagen I/III, fibronectin, hyaluronan, cross-linking enzymes, matrix stiffening | Physical exclusion of T cells and other leukocytes; reduced pore size and increased tissue resistance | Exercise may influence fibrosis indirectly via inflammation and perfusion, but tumor-specific proof is limited |
| **CAF-driven immune exclusion** | Cancer-associated fibroblasts | CAF secretion of CXCL12, TGF-β, matrix proteins, and contractile mediators | Creates a stromal barrier that traps CXCR4+ lymphocytes at the margin | Exercise could theoretically reduce CAF-supportive inflammatory tone, but this remains hypothesis-generating |
| **Elevated interstitial fluid pressure** | Tumor interstitium, vasculature | Fluid accumulation, vessel compression, impaired drainage | Reduces convective transport and prevents stable leukocyte entry and ligand distribution | Exercise-related changes in perfusion may modulate local pressure dynamics, but direct evidence is weak |
| **Hypoxia-driven suppression** | Tumor cells, myeloid cells, endothelial cells | HIF-dependent pathways, VEGF induction, altered chemokine transcription | Promotes abnormal vasculature, myeloid recruitment, and immune exclusion | Exercise may improve systemic and local oxygen delivery, potentially attenuating hypoxia-linked suppression |
| **Acidosis and lactate accumulation** | Tumor cells, stromal cells, infiltrating leukocytes | Low pH, elevated lactate, proton-coupled metabolic stress | Impairs T-cell motility, cytoskeletal dynamics, and chemokine responsiveness | Acute exercise changes in lactate are physiologic and transient; they should not be conflated with tumor lactate biology |
| **Adenosine-rich suppressive milieu** | Tumor cells, hypoxic stromal regions | CD39/CD73-mediated adenosine generation, A2A receptor signaling | Dampens T-cell activation, migration, and cytotoxic function | Exercise may influence purinergic tone indirectly through improved perfusion and inflammation control |
| **Myeloid-derived suppression** | TAMs, neutrophils, MDSCs | CCL2, CXCL8, IL-10, VEGF, ROS, arginase, proteases | Reinforces vascular dysfunction, ECM remodeling, and immune exclusion | Exercise may shift systemic myeloid balance, but tumor-level effects require direct validation |
| **Failure of rolling and firm adhesion** | Leukocytes and endothelium | Insufficient selectin-mediated rolling, reduced integrin activation, low ICAM-1/VCAM-1 availability | Cells remain intravascular or fail to arrest efficiently on tumor vessels | Exercise may improve leukocyte mobilization and endothelial activation, but homing remains barrier-limited |
| **Impaired transmigration** | Leukocytes, endothelial junctions | Junctional disorganization, abnormal permeability, low permissiveness of tumor vessels | Leukocytes arrest but fail to cross into tissue | Vascular normalization induced by training may improve transendothelial migration |
| **Post-extravasation mechanical entrapment** | T cells, NK cells, myeloid cells | Dense matrix, narrow stromal pores, increased tissue stiffness | Cells enter tissue but cannot penetrate tumor nests; immune exclusion persists | Exercise may influence stromal remodeling over time, but evidence remains mostly inferential |
| **Suppressed receptor competence** | Circulating and tissue-infiltrating leukocytes | Desensitization, altered receptor density, inflammatory cross-talk, biased signaling | Cells fail to respond properly even when chemokines are present | Systemic training may modify leukocyte phenotype, but receptor-specific tumor trafficking effects are not yet established |
| **Coupled barrier amplification** | Whole TME | Hypoxia → VEGF → vessel abnormality → more hypoxia; fibrosis → compression → poor perfusion → more hypoxia | Self-reinforcing exclusion loop that resists single-target interventions | Exercise may be relevant because it can act on perfusion, inflammation, and systemic immune fitness simultaneously |

# Table S6. Putative Mechanisms by Which Exercise Remodels the Tumor Chemokine Landscape

| **Regulatory Level / Process** | **Exercise-Responsive Change** | **Candidate Chemokine Axes / Mediators** | **Predicted Consequence for Immune Trafficking** | **Current Strength / Interpretation of Evidence** |
| --- | --- | --- | --- | --- |
| **Systemic leukocyte mobilization** | Acute exercise redistributes NK cells, CD8+ T cells, and other effector populations through catecholaminergic and hemodynamic signaling | Indirect effects on trafficking responsiveness rather than tumor-specific ligand induction | Increases circulating availability of effector cells, but does not by itself ensure tumor entry | Strong for systemic mobilization; insufficient alone as evidence of improved intratumoral homing |
| **Interferon-linked chemokine induction** | Exercise may enhance IFN-associated inflammatory programming in tumors or host immune compartments | **CXCL9/CXCL10/CXCL11–CXCR3** | Favors recruitment of activated effector T cells and supports transition toward a more inflamed phenotype | Currently the most biologically plausible and comparatively best-supported axis |
| **Myeloid remodeling** | Repeated exercise may alter macrophage polarization and reduce suppressive myeloid accumulation | CCL2, CXCL8, CCL5, myeloid-associated inflammatory mediators | May reduce chemokine programs that favor suppressive recruitment and vascular dysfunction | Moderate but context-dependent; often indirect and model-sensitive |
| **Vascular normalization / endothelial permissiveness** | Exercise may improve perfusion, endothelial phenotype, and adhesion molecule support | Endothelial chemokine display; ICAM-1/VCAM-1-linked recruitment interfaces | Improves rolling, arrest, adhesion, and transendothelial migration; may increase functional accessibility of chemokines | Moderately supported in exercise-oncology models; mechanism plausible but not uniform |
| **Stromal remodeling** | Exercise may alter stromal organization, fibroblast activity, and matrix density | **CXCL12–CXCR4**, CAF-derived chemokine retention fields | May weaken stromal trapping at invasive margins and improve parenchymal penetration | Promising but still preliminary; direct evidence remains limited |
| **Metabolic reprogramming of the TME** | Exercise may attenuate hypoxia, improve oxygen delivery, and modify inflammatory-metabolic stress | Hypoxia-linked chemokine regulation, lactate-sensitive inflammatory signaling, adenosine-associated suppression | Restores the conditions in which chemokine gradients can be productively interpreted by effector cells | Mechanistically compelling, but much of the evidence is inferential rather than axis-resolved |
| **Shift in chemokine hierarchy** | Exercise may change the balance between effector-recruiting versus suppressive/retention-associated signals | CXCR3 ligands versus CXCL12-, CCL2-, or CXCL8-dominant programs | Moves tumors from immune-deserted or immune-excluded states toward more infiltration-permissive states | Useful as an integrative framework; should be presented as a model rather than a settled conclusion |
| **Cellular re-composition of the TME** | Exercise may change the abundance or state of endothelial cells, fibroblasts, macrophages, and lymphocytes that produce or interpret chemokines | Multi-axis; includes CXCR3, CCR5, CXCR4-related signaling environments | Alters both ligand production and receptor-guided cellular behavior within the same tissue | Supported conceptually and in some preclinical studies, but heterogeneity across tumors is substantial |
| **CCR5-associated remodeling** | Exercise may affect inflammatory programs linked to lymphocyte and myeloid trafficking | **CCL5–CCR5** | Could support cytotoxic recruitment in some settings, but may also sustain inflammatory or prometastatic programs in others | Context-dependent and not uniformly beneficial; requires careful wording |
| **Epigenetic or transcriptional persistence** | Repeated exercise may induce durable changes in inflammatory set-points or chemokine-related transcriptional programs | Interferon-responsive genes, chromatin-linked persistence mechanisms | May help sustain a more permissive chemokine environment beyond acute exercise bouts | Hypothesis-generating; attractive but still relatively early |
| **Dose / timing dependence** | Effects differ by exercise intensity, duration, chronicity, and sampling window | All axes potentially affected in time-sensitive fashion | Same intervention may appear beneficial, neutral, or misleading depending on when tissue is sampled | Important interpretive principle; strongly justified even where mechanistic proof is incomplete |
| **Tumor-context conditioning** | Exercise effects depend on tumor type, vascular phenotype, stromal density, and baseline immune contexture | Axis-specific conditioning across CXCR3, CCR5, and CXCR4 systems | Explains why not all tumors respond with improved infiltration or identical chemokine remodeling | Essential caveat; should be stated explicitly to avoid overgeneralization |

Table S6. Mechanistic framework for exercise-mediated remodeling of the tumor chemokine landscape. The table summarizes the principal regulatory levels through which exercise may influence chemokine production, presentation, accessibility, and interpretation within the tumor microenvironment, with emphasis on candidate chemokine axes, trafficking consequences, and the current strength of evidence. Abbreviations CAF: Cancer-Associated Fibroblast ICAM-1: Intercellular Adhesion Molecule-1 IFN: Interferon NK: Natural Killer TME: Tumor Microenvironment VCAM-1: Vascular Cell Adhesion Molecule-1

# Table S7. The CXCL9/CXCL10/CXCL11–CXCR3 Axis: Molecular Checkpoints and Exercise-Mediated Restoration of Antitumor Homing

| Component / Mechanistic Level | Role in Tumor Immunity & Trafficking | Exercise-Relevant Regulatory Mechanism | Predicted Consequence for Immune Infiltration | Current Strength & Evidentiary Boundaries |
| --- | --- | --- | --- | --- |
| CXCR3 Receptor Expression | Prominently expressed on activated CD8+ T cells, NK cells, and Th1 lymphocytes; required for directional sensing. | Acute exercise-induced catecholamine spikes preferentially mobilize CXCR3+ effector subsets into the circulation. | Increases the systemic pool of ‘trafficking-ready’ effector cells available for tumor recruitment. | Strong: High certainty regarding systemic mobilization of CXCR3+ cells in humans and mice. |
| Upstream IFN-$\gamma$ Signaling | Central driver for the transcriptional induction of CXCL9, CXCL10, and CXCL11 in myeloid and stromal cells. | Exercise can transiently elevate systemic and local IFN-$\gamma$ levels, possibly via NK cell and T-cell activation. | Enhances the ‘Type-1’ inflammatory tone, favoring the production of CXCR3-specific ligands over suppressive ones. | Moderate: Plausible link; IFN-$\gamma$ is exercise-responsive, but direct intratumoral induction is context-dependent. |
| Endothelial Luminal Presentation | CXCL9/10 must be immobilized on GAGs at the vascular interface to trigger leukocyte integrin activation. | Training-induced vascular normalization may improve the spatial display and density of chemokines on the endothelium. | Converts ‘rolling’ leukocytes into ‘adherent’ ones; acts as the primary gatekeeper for vascular exit into the TME. | Mechanistic/Plausible: Supported by vascular biology; direct exercise-oncology proof is still emerging. |
| Transendothelial Migration (TEM) | CXCR3 engagement is indispensable for leukocytes to cross the tumor endothelial barrier (as seen in melanoma). | Improved perfusion and reduced endothelial anergy (via lower VEGF/ET-1) enhance the efficiency of TEM. | Ensures that mobilized cells actually enter the parenchyma instead of remaining trapped in the vasculature. | Strong (Biological): CXCR3 is a proven TEM checkpoint. Preliminary (Exercise): Needs more direct validation. |
| Hypoxia & HIF-1$\alpha$ Interference | Hypoxia distorts endothelial cues and promotes suppressive recruitment (e.g., via CCL2/CXCL8). | Exercise attenuates hypoxia and reduces HIF-1$\alpha$ stabilization through improved oxygen delivery. | Redirects the chemokine hierarchy away from myeloid-attracting cues toward CXCR3-dependent cytotoxic cues. | Moderate/High: Compelling evidence that exercise alleviates hypoxia and shifts immune phenotypes. |
| Spatial Routing & Haptotaxis | Guides T cells from the perivascular space into deep tumor nests (parenchymal infiltration). | Altered interstitial fluid pressure and ECM remodeling may stabilize the haptotactic CXCL9/10 gradients. | Promotes a transition from an ‘immune-excluded’ (cells at margin) to an ‘inflamed’ (cells in nest) phenotype. | Inferential: Based on tissue organization principles; direct imaging in exercise models is needed. |
| Myeloid-to-Effector Ratio | Imbalance in CXCR3 ligands vs. suppressive chemokines dictates the ‘immune contexture’. | Repeated exercise may repolarize TAMs and MDSCs, favoring their production of CXCL9/10. | Shifts the TME from a wound-healing/suppressive state to a cytotoxic-permissive inflammatory state. | Moderate: Supported by preclinical models showing myeloid remodeling with training. |
| Translational Synergy (e.g., ICB) | CXCR3 axis is required for the efficacy of anti-PD-1/PD-L1 therapies. | Exercise may act as a ‘priming’ stimulus that restores the trafficking syntax needed for ICB-responsive cells. | Potentiates immunotherapy by ensuring that therapy-induced T cells can reach their malignant targets. | High (Translational): Important for combination therapy design; highly relevant for clinical trials. |
| Clinical Evidentiary Boundary | Most data on this axis come from preclinical models or systemic human sampling. | Human intratumoral biopsy data following exercise are scarce. | Caution against claiming universal “normalization” across all human cancer types and stages. | Essential Limitation: Direct human intratumoral chemokine quantification is currently limited. |

Abbreviations: ET-1: Endothelin-1 GAG: Glycosaminoglycan HIF-1 𝛼 α : Hypoxia-Inducible Factor 1-alpha ICB: Immune Checkpoint Blockade IFN- 𝛾 γ : Interferon-gamma MDSC: Myeloid-Derived Suppressor Cell TAM: Tumor-Associated Macrophage TEM: Transendothelial Migration TME: Tumor Microenvironment VEGF: Vascular Endothelial Growth Factor

# Table S8. Context-Dependent Remodeling of the CCL5–CCR5 Axis by Exercise

| Mechanistic level | Antitumor function of CCL5–CCR5 | Protumor / suppressive function of CCL5–CCR5 | Exercise-relevant modulation | Predicted consequence for tumor immune trafficking | Evidence strength / boundary |
| --- | --- | --- | --- | --- | --- |
| CCR5 on activated CD8+ T cells and NK cells | Supports directional homing of cytotoxic lymphocytes to inflamed tumor tissue | If effector cells are numerically or functionally depleted, ligand signaling becomes less productive | Acute exercise mobilizes leukocytes and may increase circulating effector-cell availability | Greater probability that available CCR5+ effector cells can respond to tumor-derived CCL5 | Moderate to strong for exercise-induced leukocyte mobilization; limited for direct tumor-specific CCR5 remodeling |
| CCL5 as chemotactic ligand | Functions as a signpost for effector-cell recruitment and intratumoral localization | Can also attract suppressive CCR5+ myeloid cells depending on cellular context | Exercise may not directly change CCL5 abundance but may alter the functional meaning of an existing gradient | Axis output depends on receptor distribution and tissue accessibility rather than ligand level alone | Strong conceptual support, but direct human intratumoral evidence remains limited |
| Endothelial activation and adhesion molecules | Promotes rolling, firm adhesion, and transendothelial migration when the vascular interface is receptive | Endothelial dysfunction can prevent productive decoding of CCL5 signals | Exercise may improve endothelial function, perfusion, and vascular accessibility | Makes CCR5-mediated recruitment biologically executable rather than merely theoretical | Moderate; biologically plausible, but exercise-oncology linkage is still indirect |
| Vascular normalization / perfusion | Better oxygen delivery and flow increase immune-cell access to tumor vessels | Poor perfusion and abnormal vasculature can trap immune cells in circulation or at the periphery | Exercise may partially normalize tumor vasculature and reduce flow limitation | Increases the chance that CCR5+ effector cells reach the vascular checkpoint | Moderate, stronger for exercise effects on perfusion than for CCL5-specific readouts |
| Hypoxia / HIF-1α-linked dysfunction | Reduced hypoxia may favor immune-permissive trafficking conditions | Hypoxia can sustain suppressive chemokine programs and myeloid recruitment | Exercise may attenuate hypoxia through better oxygenation and vascular remodeling | Shifts the TME toward a context in which CCR5-driven trafficking can be productive | Moderate for hypoxia attenuation; inferential for CCL5-specific consequences |
| Myeloid-derived suppressor cells (MDSCs) | Not an antitumor component of the axis | CCL5 can recruit CCR5+ MDSCs and reinforce immune suppression | Exercise may reduce suppressive polarization indirectly, but this is not proven as a CCL5-specific effect | If suppressive myeloid recruitment falls, effector-cell dominance becomes more likely | Limited to moderate; strong mechanistic rationale, weak direct exercise-tumor proof |
| Tumor-associated macrophages (TAMs) | Can support antitumor immunity if reprogrammed toward inflammatory phenotypes | CCL5 may also support recruitment/maintenance of protumor TAMs | Exercise may shift macrophage polarization away from a wound-healing phenotype | Could alter the net output of the CCL5–CCR5 axis toward less suppression | Moderate for exercise-related macrophage reprogramming; context dependent |
| Inflammatory tone (IL-6, IFN-γ, catecholamines) | Can support cytotoxic recruitment and immune activation when coordinated appropriately | Excess or dysregulated inflammation may still favor myeloid recruitment | Exercise changes systemic inflammatory and neuroendocrine signaling, potentially changing the “reading” of CCL5 | Alters whether the pathway behaves like an effector-cell recruitment program or a suppressive routing system | Moderate, but direct causal mapping to CCL5 in humans is not established |
| Acute exercise vs chronic training | Acute bouts may transiently increase trafficking-ready lymphocytes; chronic training may improve tissue permissiveness | If the tumor remains myeloid-dominant, exercise effects may be blunted | Acute exercise = mobilization; chronic exercise = milieu remodeling | Acute and chronic exercise may act at different points of the trafficking cascade | Strong conceptual distinction; important to state explicitly |
| Vascular/stromal accessibility | Enables chemokine decoding at the endothelial interface | Dense stroma and poor accessibility can prevent effective infiltration despite ligand presence | Exercise may improve stromal permeability indirectly via oxygenation and perfusion changes | Enhances the probability that CCR5+ cells can enter the parenchyma | Inferential, but highly relevant mechanistically |
| Immune contexture / receptor distribution | Favorable when CCR5 is enriched on cytotoxic lymphocytes | Unfavorable when CCR5 is enriched on suppressive myeloid populations | Exercise may shift systemic immune composition, but not necessarily intratumoral receptor hierarchy | Determines whether CCL5 acts as a pro-immune or pro-suppressive signal | Critical boundary variable; often ignored in simplistic reviews |
| Combination therapy relevance | May improve response to immunotherapy if effector cells can reach the tumor | May be ineffective if suppressive recruitment remains dominant | Exercise could serve as a permissive adjunct to checkpoint blockade or adoptive cell therapy | Enhances the likelihood that therapy-induced or therapy-expanded cells can home to tumor tissue | Translationally important, but still hypothesis-generating |

Table S8. Context-dependent biology of the CCL5–CCR5 axis in tumor immunity and the exercise-related factors that may alter its net trafficking output. The table distinguishes antitumor effector-cell recruitment from protumor myeloid recruitment and emphasizes that exercise is more plausibly a permissive contextual modifier than a direct regulator of CCL5 abundance.

# Table S9. The CXCL12–CXCR4 Axis as a Retention and Immune-Exclusion Program: Exercise-Relevant Mechanisms and Evidentiary Boundaries

| Component / mechanistic level | Role in CXCL12–CXCR4-mediated exclusion | Exercise-relevant regulatory mechanism | Predicted consequence for immune trafficking | Current strength and evidentiary boundary |
| --- | --- | --- | --- | --- |
| CXCL12 production by CAFs, endothelial cells, and tumor cells | Generates spatially restricted chemokine niches that can retain leukocytes in stromal or perivascular compartments rather than support access to malignant nests. | Chronic exercise may modify the hypoxic, fibrotic, and inflammatory conditions that sustain CXCL12-producing stromal programs. | May reduce the functional dominance of stromal retention signals, even without complete suppression of CXCL12 expression. | Strong for stromal CXCL12 biology; limited for direct exercise-induced reduction of intratumoral CXCL12. |
| CXCR4 expression on immune and progenitor cells | Directs the migration, retention, and compartmental positioning of CXCR4-expressing leukocytes in response to CXCL12 gradients. | Exercise alters systemic immune composition and activation states, potentially changing the abundance or responsiveness of CXCR4-expressing cellular readers. | May modify how the existing CXCL12 field is interpreted by immune cells. | Plausible but indirect; receptor-level effects in human tumor tissue require direct measurement. |
| Stromal sequestration of CD8+ T cells | Promotes accumulation of immune cells within peripheral stroma, fibroblast-rich areas, or perivascular regions without productive tumor-cell contact. | Exercise-associated improvements in perfusion, oxygenation, and stromal permissiveness may weaken the tissue conditions favoring sequestration. | Greater probability of movement beyond stromal boundaries toward tumor parenchyma. | Strong for the exclusion concept; hypothesis-generating for exercise-mediated release from stromal trapping. |
| Desmoplasia and extracellular-matrix architecture | Dense extracellular matrix and activated fibroblasts reinforce CXCL12-rich exclusionary barriers and mechanically constrain lymphocyte penetration. | Repeated exercise may indirectly influence matrix remodeling through altered perfusion, hypoxia, inflammatory signaling, and fibroblast activation. | May reduce physical and chemotactic barriers to intratumoral immune-cell movement. | Mechanistically credible, but direct evidence remains sparse and tumor-type dependent. |
| Matrix-bound chemokine presentation | CXCL12 can be immobilized within extracellular matrix and displayed locally, producing gradients not reflected by circulating concentrations. | Exercise-related changes in tissue perfusion and matrix turnover could alter ligand accessibility or gradient geometry. | May change local retention strength independently of total circulating CXCL12. | Critical limitation: systemic CXCL12 measurements cannot substitute for tissue-level spatial analysis. |
| Hypoxia and HIF-1α-linked stromal dysfunction | Hypoxia promotes fibroblast activation, abnormal vascularity, extracellular-matrix remodeling, and exclusionary immune architecture. | Exercise may improve oxygen delivery and reduce hypoxia-associated HIF-1α signaling. | Weakens environmental conditions that permit CXCL12-driven retention and immune exclusion to dominate. | Moderate for exercise-associated hypoxia attenuation; indirect for CXCL12-specific consequences. |
| Abnormal tumor vasculature | Dysfunctional vessels restrict immune-cell access and may reinforce perivascular accumulation rather than effective transmigration. | Exercise may improve blood flow, vascular maturity, and endothelial competence. | Increases the likelihood that mobilized immune cells reach and cross the tumor vascular interface. | Moderate for vascular effects; not specific proof of CXCL12–CXCR4 remodeling. |
| Suppressive myeloid-cell positioning | CXCL12–CXCR4 signaling can support recruitment or retention of myeloid populations that sustain immune suppression and tissue remodeling. | Exercise may reduce suppressive myeloid burden or alter macrophage polarization through repeated systemic immunometabolic adaptation. | May reduce the suppressive cellular context that reinforces retention-dominant chemokine signaling. | Limited to moderate; exercise effects vary by tumor model, intervention dose, and myeloid phenotype. |
| Competition with CXCR3-directed recruitment programs | CXCL12-mediated retention can oppose infiltration-promoting programs by maintaining immune cells in nonproductive compartments. | Exercise may strengthen interferon-linked CXCL9/CXCL10/CXCL11–CXCR3 signaling while reducing hypoxic stromal dysfunction. | Shifts the relative chemokine hierarchy toward effector-cell entry and parenchymal localization. | Conceptually strong, but requires integrated spatial profiling for direct confirmation. |
| Acute exercise | Rapid leukocyte mobilization alone does not dismantle established stromal barriers or alter matrix-bound CXCL12 niches. | Catecholamine-mediated redistribution transiently expands the circulating pool of trafficking-capable immune cells. | Enhances immune-cell availability but is unlikely, by itself, to overcome retention or exclusion. | Strong distinction: systemic mobilization is established; local release from CXCL12 barriers is unproven. |
| Chronic exercise training | Repeated exposure may progressively modify vascular function, inflammatory tone, hypoxia, stromal organization, and systemic chemokine set points. | Training-related adaptation provides the more plausible route for altering the functional context of CXCL12–CXCR4 signaling. | May gradually soften retention-dominant tissue logic and improve responsiveness to infiltration-promoting cues. | Biologically plausible; longitudinal tumor-biopsy studies are needed. |
| Systemic versus intratumoral CXCL12 | Circulating CXCL12 reflects only one compartment and may not represent fibroblast-anchored or matrix-bound intratumoral ligand gradients. | Exercise-associated changes in blood CXCL12 should be interpreted as systemic modulation, not proof of local tumor remodeling. | Prevents overinterpretation of systemic biomarker changes as evidence of restored intratumoral trafficking. | Essential evidentiary boundary for all exercise-oncology interpretations. |
| Combination-treatment relevance | CXCL12–CXCR4-mediated exclusion may limit the efficacy of therapies that activate or expand T cells but do not resolve stromal access barriers. | Exercise could act as a physiological adjunct alongside immune checkpoint blockade, adoptive cell therapy, CXCR4 antagonism, or vascular-normalizing strategies. | May improve the probability that therapy-activated effector cells reach malignant tissue. | High translational relevance, but remains a testable combination hypothesis rather than established clinical practice. |

# Table S10. Acute versus Chronic Exercise as Distinct Regulatory Programs in Chemokine Biology: Signaling, Transcriptional, Epigenetic, and Trafficking-Relevant Adaptations

| Regulatory domain | Acute exercise: dominant biology | Chronic exercise training: dominant biology | Relevance to chemokine regulation and immune trafficking | Exercise-oncology interpretation / evidentiary boundary |
| --- | --- | --- | --- | --- |
| Primary physiological logic | Rapid systemic perturbation; short-lived neuroendocrine, hemodynamic, and immunological flux. | Progressive adaptation; establishment of new vascular, inflammatory, and immunometabolic set points. | Distinguishes transient immune-cell availability from durable remodeling of the signaling context in which trafficking occurs. | Core interpretation: acute exercise mainly mobilizes; chronic training may remodel. |
| Time scale of effect | Minutes to hours after a single bout. | Days to weeks to months with repeated exposure. | Chemokine changes after acute exercise are typically too brief or small to reflect stable network reorganization, whereas repeated training may generate detectable resting-state shifts. | Prevents conflation of immediate leukocytosis with sustained chemokine remodeling. |
| Dominant upstream drivers | Catecholamines, glucocorticoids, myokines (especially IL-6), shear stress, transient metabolic stress. | Recurrent exposure to these same signals, but with cumulative adaptation across endothelial, immune, stromal, and metabolic compartments. | Acute drivers predominantly redistribute cells; chronic repetition may gradually recalibrate the regulatory circuits controlling chemokine expression and receptor responsiveness. | Same mediators are involved, but not with the same biological meaning across time. |
| Main immune consequence | Transient leukocytosis and rapid mobilization of NK cells, activated CD8+ T cells, and other trafficking-competent populations. | More stable reconditioning of immune composition, receptor readiness, inflammatory tone, and trafficking competence. | Acute exercise increases the circulating supply of potential tumor-infiltrating cells; chronic training may improve the ability of those cells to interpret and use chemotactic cues. | Acute = availability; chronic = permissiveness plus competence. |
| Effect on circulating chemokines | Usually limited, inconsistent, or not clearly sustained after a single session. | More plausible route to measurable resting changes in systemic chemokines such as CCL2 and CXCL12 after repeated training. | Supports the idea that chronic exercise can shift inflammatory set points, whereas acute exercise usually does not produce a durable chemokine signature. | Systemic changes are informative but remain indirect. |
| Signaling architecture | Short-lived activation of stress-responsive pathways with rapid onset and resolution. | Repeated oscillatory exposure may entrain more stable signaling thresholds and context-dependent responsiveness. | Particularly relevant for chemokine systems governed by NF-$\kappa$B, STATs, HIF-1$\alpha$, interferon-responsive factors, and stress-sensitive transcriptional nodes. | Chronic training is more likely than a single bout to alter baseline pathway dominance. |
| Transcriptional regulation | Transient perturbation of inflammatory and trafficking-related transcriptional programs. | Cumulative resetting of basal gene-expression tendencies across immune, vascular, and stromal compartments. | Offers a plausible mechanism for resting-state changes in chemokine tone after training, even when acute sessions show minimal measurable effect. | Strong mechanistic logic; direct tumor-resolved proof remains limited. |
| Epigenetic adaptation | Unlikely to induce durable molecular memory after a single bout in the tumor-trafficking context. | Repeated training may influence DNA methylation, chromatin accessibility, histone marks, and enhancer activity across relevant tissues. | Provides a biologically coherent explanation for persistence of altered inflammatory and chemotactic set points beyond the final exercise bout. | Important caution: plausible explanatory layer, not established proof for intratumoral chemokine loci. |
| Endothelial phenotype | Temporarily altered flow, shear stress, and leukocyte-endothelium encounter frequency. | Progressive improvement in endothelial competence, inflammatory sensing, adhesion support, and transmigration permissiveness. | Chronic exercise may improve the vascular interface required for chemokine-guided entry into tissue. | Stronger rationale for chronic than acute effects on trafficking efficiency. |
| Stromal and matrix context | Minimal immediate effect on fibroblast programs, matrix architecture, or hypoxia-linked exclusion. | Repeated training may gradually alter hypoxia, perfusion, stromal activation, and matrix conditions that shape chemokine immobilization and exclusion. | Relevant for whether immune cells merely circulate near the tumor or actually penetrate into parenchyma. | Acute mobilization does not equal release from stromal trapping. |
| Hypoxia and metabolic stress | Short-term perturbation without established local reversal of tumor hypoxia. | More plausible capacity to attenuate systemic and tissue conditions that sustain HIF-linked inflammatory and exclusionary programs. | May indirectly influence chemokines embedded in hypoxic and stromal inflammatory circuits, including retention-associated signals. | Exercise should not be framed as a direct acute suppressor of tumor hypoxia-driven chemokines. |
| Chemokine receptor readiness | Increased immediate exposure of circulating cells to mobilizing signals, but limited evidence for stable receptor reprogramming after one bout. | Repeated training may affect receptor abundance, migratory polarization, and responsiveness to chemotactic gradients. | Trafficking depends not only on ligand abundance but also on whether leukocytes are competent readers of the spatial code. | Strong conceptual value; needs direct receptor-level validation in oncology settings. |
| Tumor microenvironment implication | Raises the probability of immune–tumor encounter by increasing vascular patrol of effector cells. | May progressively alter the permissiveness of the tumor-host interface through vascular, inflammatory, stromal, and transcriptional remodeling. | Captures the difference between a temporary increase in “cell supply” and a deeper change in “tissue rules.” | This is the central acute-versus-chronic distinction for the review. |
| Expected therapeutic meaning | Best understood as a short-window mobilization strategy. | Best understood as a remodeling strategy that may support repeated immune access and better integration with therapy. | Chronic training is more likely to potentiate endogenous chemokine gradients and treatment responsiveness than isolated bouts alone. | Translationally strong, but still requires direct tumor-based confirmation. |
| Measurement caveat | Post-bout serum snapshots mainly reflect systemic flux and redistribution. | Resting-state serum changes after training may indicate recalibration, but still do not directly define intratumoral gradients. | Serum biomarkers cannot substitute for tumor-resolved spatial chemokine mapping, stromal localization, or receptor-specific tissue analysis. | Essential evidentiary boundary for the entire section. |
| Most rigorous summary | Mobilization strategy: increases transient immune-cell availability and vascular surveillance. | Remodeling strategy: may durably modify the inflammatory, vascular, transcriptional, and potentially epigenetic conditions governing trafficking. | Integrates the temporal biology of exercise with the molecular syntax framework of the TME. | Best reviewer-safe formulation for this literature. |

Table S10. Acute and chronic exercise should be interpreted as biologically distinct regulatory programs in chemokine-guided immune trafficking. Acute exercise primarily increases transient immune-cell mobilization and vascular availability, whereas chronic training more plausibly contributes to durable remodeling of inflammatory tone, endothelial competence, chemokine regulation, and potentially transcriptional and epigenetic set points. Systemic biomarkers remain informative but do not constitute direct evidence of intratumoral chemokine remodeling.
